# Insights into endometriosis symptom trajectories and assessment of surgical intervention outcomes using longitudinal actigraphy

**DOI:** 10.1038/s41746-025-01629-8

**Published:** 2025-05-02

**Authors:** Katherine Edgley, Philippa T. K. Saunders, Lucy H. R. Whitaker, Andrew W. Horne, Athanasios Tsanas

**Affiliations:** 1 Centre for Reproductive Health, Institute for Regeneration and Repair, University of Edinburgh, Edinburgh, UK; 2 Usher Institute, University of Edinburgh, Edinburgh, UK

**Keywords:** Chronic pain, Fatigue, Scientific data, Outcomes research, Biomedical engineering

## Abstract

Endometriosis is a common, chronic condition associated with debilitating pain, fatigue, and heterogeneous symptom presentation. In this exploratory study, 68 participants with confirmed endometriosis were monitored for up to three 4–6-week smartwatch cycles. We collected daily self-reports of pain and fatigue as well as retrospective questionnaires assessing quality of life, and we extracted daily measures of physical activity (PA), sleep, and diurnal rhythms from wrist-worn actigraphy data. We found that daily PA was strongly negatively correlated with self-reported fatigue (repeated measures correlations $R < -0.3$) and that participants with more severe or variable symptom trajectories displayed lower levels of PA, greater sleep disturbance, and more disrupted sleep and activity rhythms (Spearman’s ${|R|} > 0.3$). Lastly, we found evidence of sleep and PA changes following surgery for endometriosis that reflected change in self-reported symptoms. Collectively, our findings suggest that passive data collection using wrist-worn wearables in endometriosis could facilitate individualized objective insights into symptom trajectories.

## Introduction

Endometriosis is a chronic condition associated with debilitating symptoms including pain and fatigue. Despite its prevalence, with reported estimates of affecting approximately 10% of women of reproductive age (190 million women globally), treatment options for managing symptoms are often unsatisfactory and diagnostic delays are common^1^. The condition is diagnosed when endometriosis lesions, or tissue like that found on the lining of the uterus (endometrium), are found outside of the uterus^2^. Lesion characteristics are generally categorized into three main subtypes: superficial peritoneal (representing approximately 80% of cases), deep, and ovarian^3,4^. Symptom presentation in endometriosis can vary widely between individuals, with some women being asymptomatic, and others experiencing life-altering symptoms such as chronic pelvic pain (either with menstruation or non-cyclical) and fatigue, as well as gastrointestinal symptoms and psychological symptoms (depression and anxiety)^4^. Typically, lesions are visualized surgically using laparoscopy, though ovarian and deep disease may be identified using imaging methods^2^.

Endometriosis lesions, when characterized through current staging systems, do not clearly correspond to symptom presentation^2,5^. Thus, endometriosis symptom severity is generally assessed using patient-reported outcome measures (PROMs), and there exist limited objective approaches to assess outcomes and changes in symptoms, such as through changes in behaviors or physiological signals^6^. Standard treatments for endometriosis include surgical (laparoscopic) removal of lesions and hormonal medications^2^. However, further methods for managing symptoms and reducing impact on quality of life (QoL), including non-medical and self-management methods, remain an important research priority^7^. Questionnaire-based studies have additionally reported that endometriosis symptoms likely have an impact on sleep^8^ and physical activity (PA)^9^, while exercise and PA-related interventions have also been investigated as potential therapeutic non-invasive approaches to mitigate symptoms^10,11^.

Digital approaches towards collecting symptom-tracking data have proved promising in recent endometriosis studies, where app-based PROMs and custom self-tracking of activities or interventions were used to inform patient phenotypes and the relationship between self-reported exercise and symptoms^12,13^. Although PROMs are useful to understand participants’ own perception of symptoms, they can be burdensome, especially when collected daily, resulting in missing data which can introduce bias, and responses can be influenced by a range of factors including mood and recall^14^. Furthermore, given that endometriosis patients often report “*flares*” or periods of elevated symptom severity, questionnaires captured retrospectively may not reflect the symptoms’ temporal resolution (how quickly symptoms change) or miss valuable information on the effect of treatments. Wearable devices offer the opportunity to passively detect objective behaviors or physiological signals, thus allowing for continuous longitudinal assessment, and have been explored in diverse health monitoring conditions^6,15,16^. However, few studies to date have utilized objective approaches to capture behaviors that may relate to endometriosis symptoms^6^.

Using *actigraphy*, the collection of three-dimensional acceleration data typically using wrist-worn accelerometers, one can passively and longitudinally collect continuous data which enable extracting sleep, PA, and diurnal rhythm patterns in a free-living environment^17–19^. In conjunction with PROMs, actigraphy data can shed light on the relationship between symptoms and objectively measured sleep and PA, as has been demonstrated by studies in other conditions^6,18,20^.

In this study, we used both actigraphy-derived and self-reported data to characterize endometriosis symptom trajectories and examine how symptoms relate to PA, sleep, and diurnal rhythms. As the first large study in endometriosis to capture both objective and subjective measures of symptoms longitudinally, we aim to present the motivation for and feasibility of using wearable-derived data in endometriosis to provide new insights into patient outcomes.

## Results

### Data collection

**Fig. 1:**
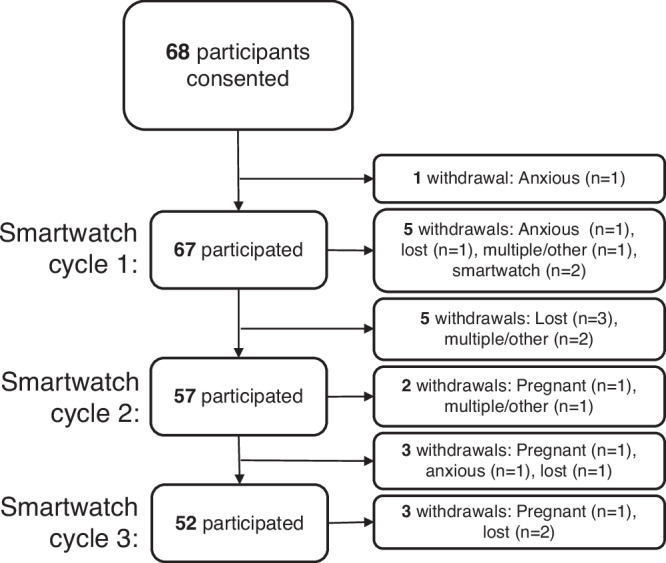
Flow chart of participation in the study for the three 4-6-week smartwatch cycles. A participant was defined to have “participated” in a smartwatch cycle if either more than one PROM was submitted or smartwatch data was returned for the smartwatch cycle. “Lost” indicates lost to follow-up.

The baseline characteristics of the 68 study participants (all of whom identified as female) are summarized in Table 1, and more details on hormonal medication and comorbidities are displayed in Supplementary Fig. 1. All participants had been previously diagnosed with endometriosis either through laparoscopy or imaging (for deep or ovarian endometriosis only). In this study, participants consented to wear smartwatches collecting actigraphy, submit daily PROMs on symptoms for up to three 4–6 week periods (henceforth referred to as *smartwatch cycles*, with number of smartwatch cycles denoted by *N* as opposed to *n* participants), and complete retrospective questionnaires at the end of each smartwatch cycle. Of the *n* = *68* participants consented, *n* = *66* had associated actigraphy data (5152 days of actigraphy data with >75% wear) and *n* = *67* had associated daily PROMs (5417 days). Further details on participation numbers for the data collected throughout each smartwatch cycle are provided in Fig. 1 and can also be found in Supplementary Note 1. Of the subset of participants (*n* = *20)* recruited to the surgical sub-study who underwent surgery to excise endometriosis, with or without additional total hysterectomy±bilateral salpingo-oophorectomy (BSO), *n* = *17* were confirmed to have deep or ovarian disease after receiving surgery, *n* = *16* of whom participated in one or more smartwatch cycles after surgery (and thus were included in further analysis of the sub-study). For these remaining participants, the third smartwatch cycle was completed approximately 4–6 months post-surgery (average of 151.5 days, ranging between 108 and 200 days post-surgery).

**Table 1:** Demographic characteristics and diagnoses of participants as provided at baseline

Cohort demographics and characteristics	
Variable	*n* (%) or Mean ± SD
Age	33.7 ± 7.4
Smoker	11 (16.2%)
Night shift work	2 (2.9%)
Nulliparous	46 (67.6%)
Duration of pelvic pain in months (*n* = 67)	155.3 ± 96.7
Analgesia taken regularly	41 (60.3%)
Opiates	18 (26.5%)
Analgesia taken for pelvic pain flares	60 (88.2%)
Opiates	36 (52.9%)
**BMI**	27.9 ± 6.4
Underweight (BMI $< 18.5$)	1 (1.5%)
Normal ($18.5\le$BMI $< 25$)	29 (42.6%)
Overweight ($25\le$BMI $< 30$)	13 (19.1%)
Obese ($30\le$BMI $< 40$)	23 (33.8%)
Severely obese (BMI $\ge 40$)	2 (2.9%)
**Ethnicity**	
White	64 (94.1%)
Asia/Asian British	1 (1.5%)
Black/African/Caribbean/Black British	0 (0%)
Multiple ethnic groups	1 (1.5%)
Other ethnic groups	2 (2.9%)
**Education** (highest achieved)	
Primary	0 (0%)
Secondary	18 (26.5%)
Tertiary	50 (73.5%)
**Endometriosis subtype**	
Superficial peritoneal	38 (55.9%)
Deep	38 (55.9%)
Ovarian	23 (33.8%)
**Hormonal medication taken**	42 (61.8%)
GnRH agonist taken – with HRT	12 (17.6%)
GnRH agonist taken – without HRT	3 (4.4%)
Combined oral contraceptive pill (COCP)	4 (5.9%)
Levonorgestrel intrauterine system (IUS)	18 (26.5%)
Progesterone only pill (POP)	6 (8.8%)
Nexplanon	3 (4.4%)
Depo-provera	1 (1.5%)
**Comorbidities**	
Fibromyalgia	6 (8.8%)
Rheumatoid arthritis	1 (1.5%)
Irritable bowel syndrome (IBS)	10 (14.7%)
Autosomal dominant polycystic kidney disease (ADPKD)	0 (0%)
Migraine	27 (39.7%)
Other arthritis	3 (4.4%)
Chronic fatigue syndrome (CFS)	4 (5.9%)
Pancreatitis	0 (0%)
Painful bladder syndrome/interstitial cystitis	5 (7.5%)
Other chronic pain	12 (17.6%)
**Gynecological history**	
Adenomyosis	9 (13.2%)
Heavy bleeding	29 (42.6%)
Fibroids	8 (11.8%)
Previous pelvic inflammatory disease (PID)	7 (10.3%)
Previous surgical treatment to endometriosis	45 (66.2%)
Previous hysterectomy	1 (1.5%)
Previous bilateral salpingo-oophorectomy	1 (1.5%)

Entries are summarized in the form mean ± standard deviation, otherwise indicating the percentage within the cohort (when using parenthesis).

### Adherence

**Fig. 2:**
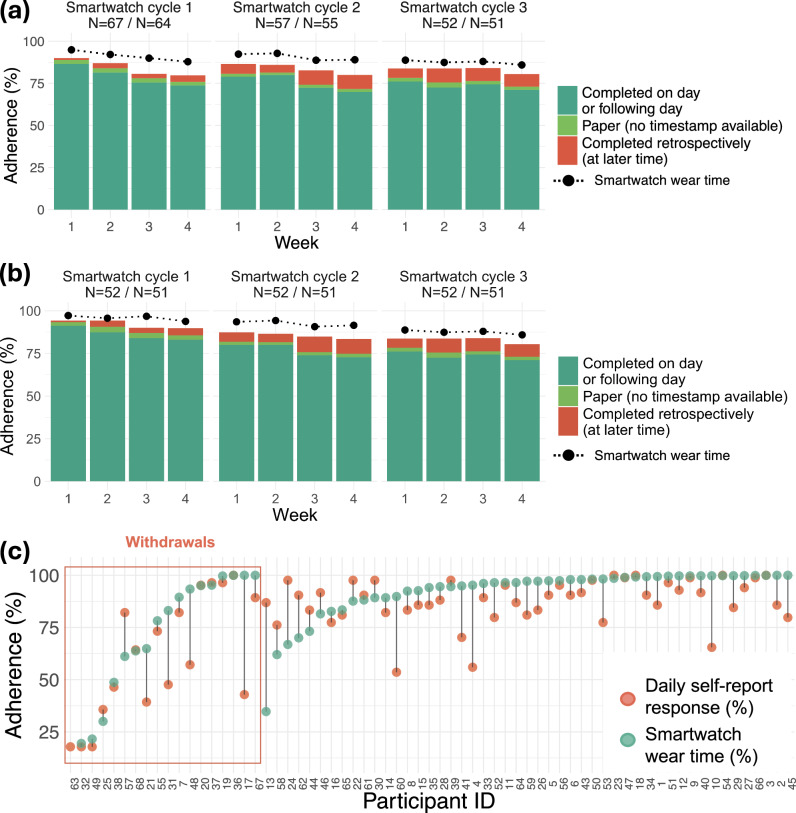
Bar plots and dot plots showing adherence patterns for the first four weeks of each smartwatch cycle and by participant. Weekly adherence patterns for the daily self-reports for the first 28 days of each smartwatch cycle are shown in (**a**) for all participants and in (**b**) for participants that participated in three smartwatch cycles. Sample size is given by smartwatch cycle (N=number of included smartwatch cycles with daily PROMs / N=number of included smartwatch cycles with returned smartwatch data). In (**c**), adherence is shown by participant, including those who withdrew as highlighted in the red box on the left. A smartwatch cycle was included if more than one PROM was submitted on day 1-28 or smartwatch data was returned. Participants that withdrew were included up until the end of the smartwatch cycle they withdrew.

Figure 2 displays the adherence levels for both PROMs and wearing smartwatches by week and by participant, showing that smartwatch adherence was generally higher than for completing PROMs, with a mean participant smartwatch wear adherence of 87.3% within the first 28 days of each smartwatch cycle compared to 80.5% for PROMs (further discussed in Supplementary Note 1). Smartwatch adherence also exhibited less decline in the fourth week of each smartwatch cycle compared to PROMs. Overall, 19 participants withdrew from the study: 16 withdrew prior to the third smartwatch cycle (three participants who withdrew during the third smartwatch cycle were thus included in Fig. 2). The reasons for withdrawal (also summarized in Fig. 1) were the following: Lost to follow-up ($n=7$), “anxious/overwhelmed” ($n=3$), both “anxious/overwhelmed” and “cannot/will not comply with daily diaries” ($n=1$), fallen pregnant ($n=3$), “didn’t want to wear the smartwatch” ($n=2$), other personal circumstances/unwell ($n=2$), and irritation from smartwatch/daily diaries increased anxiety ($n=1$).

### Exploring statistical associations within PROMs

**Fig. 3:**
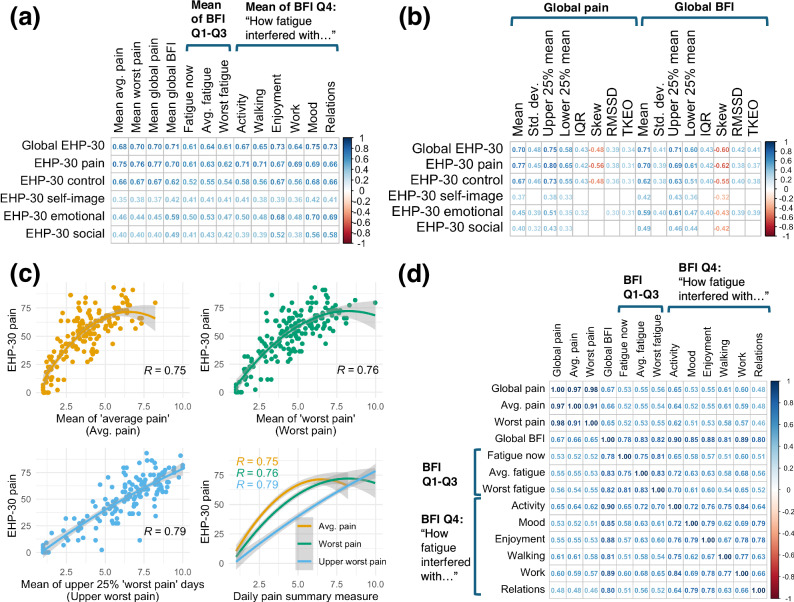
Heatmaps and scatterplots displaying correlations within PROMs. Subfigures (**a**) and (**b**) show statistically strong ( | R | > 0.3) Spearman correlations between end-of-cycle EHP-30 scores and (**a**) mean values of daily self-report questions and (**b**) summary measures of global pain and fatigue (BFI) scores. Subfigure (**c**) shows the relationship between summary measures of daily pain scores and the pain subscale of the EHP-30, depicted using Spearman correlations and overlaid best-fit quadratic curves. Subfigure (**d**) depicts repeated measures correlations within daily self-reports (using 5,349 days from *n* = 67 participants).

The correlations between end-of-cycle Endometriosis Health Profile (EHP-30) scores and daily PROMs are shown in Fig. 3 (subfigures a-b). As shown in Fig. 3a, summary measures relating to the daily average, worst, and current fatigue had lower correlations with EHP-30 scores (correlation coefficients *R* from $|0.61-0.63|$ with global EHP-30) compared to the final six questions on the Brief Fatigue Inventory (BFI) assessing the impact of fatigue on various domains (*R* ranging from $|0.64-0.75|$ with global EHP-30). Additionally, fatigue-related measures were generally more strongly associated with the emotion and social subdomains of the EHP-30 ($R=0.59$ between mean global BFI and EHP-30 emotion) compared with pain-related measures ($R=0.45$ for mean global pain and EHP-30 emotion). However, the BFI includes questions on enjoyment of life and mood, and relationships, which were most strongly correlated with similar EHP-30 domains as expected, and therefore influence the global BFI score.

Of the computed summary measures of daily global pain shown in Fig. 3b, the mean of the upper 25% of pain scores was most strongly correlated with the global EHP-30 score ($R=0.75$) as well as pain subdomain of the EHP-30 ($R=0.80$). Figure 3c shows best-fit quadratic curves to illustrate the relationship between summary measures of pain and recalled EHP-30 pain, where the mean of upper 25% of ‘worst’ pain days had the strongest correlation and most linear relationship with EHP-30 pain. These correlations suggest that the EHP-30 pain subscale may in fact reflect the most severe symptoms recalled over the previous weeks better than the average level of symptoms. Furthermore, Fig. 3b shows that variability measures of global' pain were strongly correlated with the global EHP-30 score (*R* ranging from $|0.34-0.48|$) and EHP-30 pain subdomain (*R* from $|0.31-0.45|$), which may reflect the occurrence of high pain scores, since smartwatch cycles with lower pain on average but some high pain scores would have higher variability. As shown in Fig. 3d, pairwise repeated measures correlations within the daily self-report questions revealed a very strong correlation ($R=0.67$) between daily global pain and global BFI scores, indicating that within individuals, days with severe pain symptoms reported also tended to have severe fatigue symptoms.

### Symptom trajectories and associations between daily actigraphy measures and PROMs

**Fig. 4:**
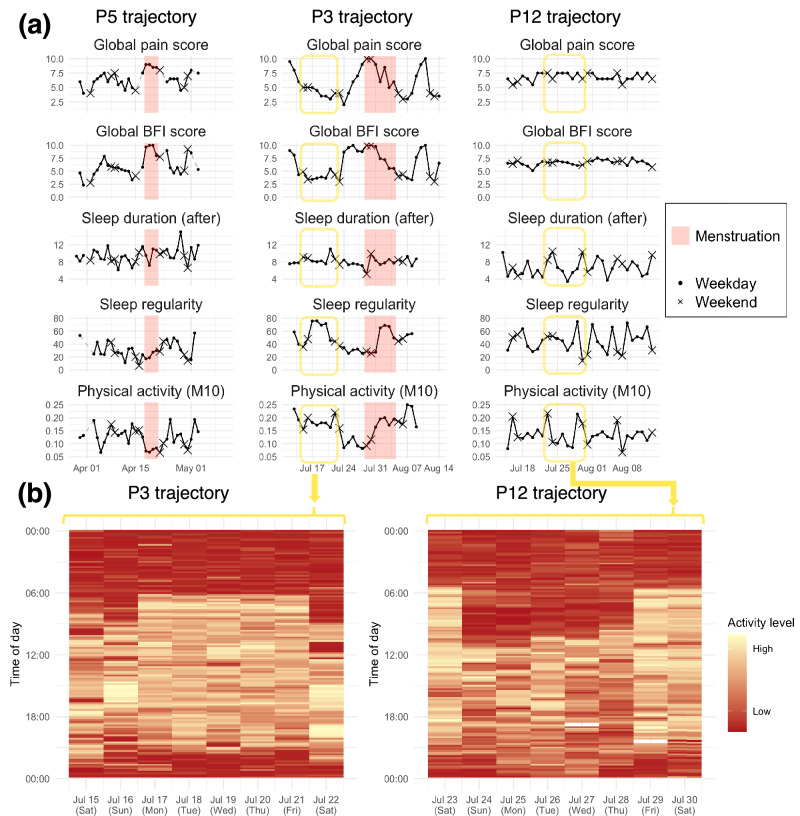
Symptom and actigraphy trajectories from indicative selected participants. **a** Examples of the trajectories of indicative participants (P5 and P3) who were not taking any hormones, compared to P12 who was taking GnRH agonist without HRT. **b** Examples of colored actograms (with log-10 transformed activity levels) of a single week from participants P3 (left, from July 15 to 22), with moderate symptoms throughout the week displayed (mean global BFI score of 4.1 and global pain score of 4.2), and P12 (right, from July 23 to 30), with severe symptoms throughout the week (mean global BFI score of 6.6 and global pain score of 7.1).

Indicative examples of symptom trajectories across a single smartwatch cycle are shown in Fig. 4. In participants with cyclical flares of pain and fatigue (P5 and P3), we found apparent changes in actigraphy-assessed sleep duration, sleep regularity, and PA that appeared to coincide with changes in self-reported pain and fatigue. In contrast, participant P12 (receiving GnRH agonist) displayed consistent and severe symptoms across the smartwatch cycle along with highly variable sleep duration and lower PA compared to participant P3 (when pain/fatigue was lower), as highlighted by the activity rhythms shown in Fig. 4b. A detailed exploration of how the severity and variability of symptoms, as well as that of daily actigraphy measures, differed by hormonal and surgical status can be found in Supplementary Figs. 5 and 6.

An example of changes in symptoms after beginning treatment with GnRH agonist is also shown in Supplementary Fig. 3, where an improvement in actigraphy-assessed PA levels and more regular sleep patterns coincided with a decrease in self-reported symptoms. However, this same relief to symptoms was not demonstrated in several participants such as P12 (Fig. 4) who were also treated with GnRH agonist and typically displayed more severe symptoms with lower variability (see Supplementary Fig. 5). Detected sleep trajectories also varied widely between participants, with some participants exhibiting abnormally long sleep (sustained inactivity) periods up to 20 hours using both sleep detection algorithms, as demonstrated in Supplementary Fig. 2, with an indicative example in Supplementary Fig. 9. A plausible explanation for this observation likely reflects that participants were lying on a bed or sofa for a prolonged time, which we can reasonably attribute to excessive fatigue or other pain.

Repeated measures correlations, which illustrate ‘within-person’ correlations, between daily actigraphy measures and PROMs indicated that certain actigraphy-assessed PA and diurnal rhythm measures were strongly correlated (${|R|} > 0.3$) with self-reported fatigue (see Supplementary Data 1 for all correlations, which were computed using all available data from $n=66$ participants, with a minimum of 3589 and maximum of 4521 degrees of freedom). Ratings of how fatigue interfered with ‘activity’ and ‘work’ (BFI Q4) were most strongly correlated with PA. Relative amplitude of the most and least active hours (RA) was the actigraphy measure most strongly correlated with fatigue, although other average PA measures such as movement during the most active 10 hours (M10) were also strongly correlated with the global BFI score. In general, repeated measures correlations between actigraphy measures and self-reported pain (*R* up to $|0.23|$) were lower when compared to fatigue (*R* up to $|0.35|$), and importantly, none of the daily actigraphy measures were strongly (${|R|} > 0.3$) correlated with self-reported pain.

**Fig. 5:**
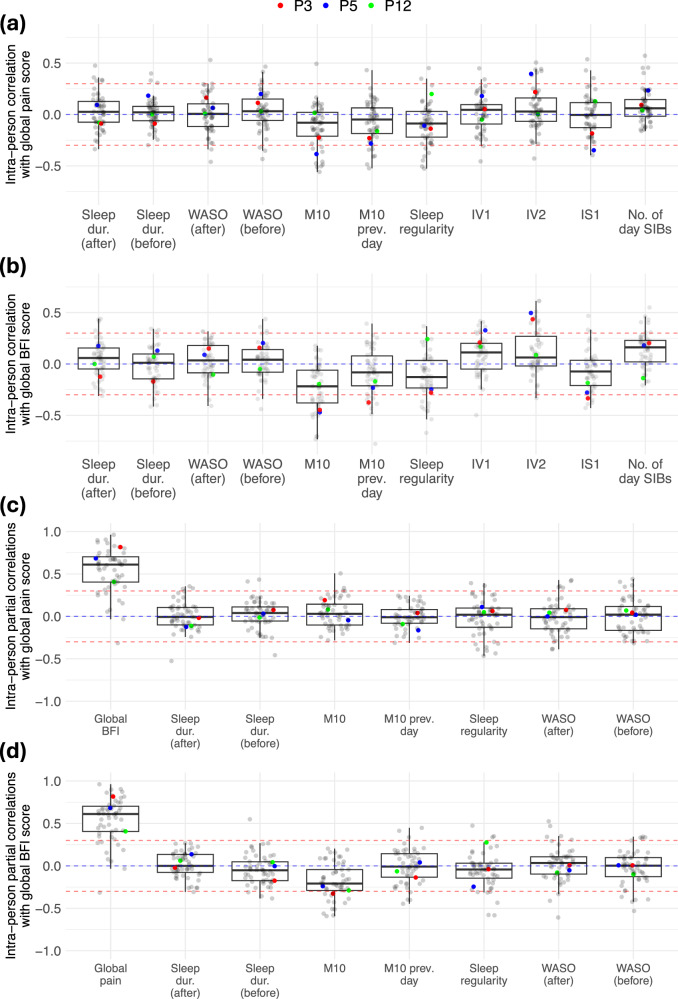
Boxplots of intra-person correlations between actigraphy measures and self-reported symptoms. Boxplots of intra-person Pearson pairwise (**a**, **b**) and partial (**c**, **d**) correlations between a range of actigraphy measures (x-axis) and (**a**, **c**) global pain scores, or (b,d) global BFI scores. In (**c**) and (**d**), the partial correlations indicate that all the other variables presented are controlled for when computing each individual correlation, as opposed to (**a**) and (**b**) where pairwise correlations do not control for any variables. Each point represents the correlation within a specific participant’s own data, and red-dashed lines indicate where |R | =0.3. We refer to Fig. 9 and Supplementary Data 1 for definitions of variable names. Only participants with at least 20 non-missing value pairs were included, and smartwatch cycles in which participants received surgery for endometriosis (either as part of the surgical sub-study or otherwise) were excluded, resulting in *n* = 54 participants included in the figure, with a total number of days included ranging from 3,591-3,907 for subfigures (**a**) and (**b**) and 3,327 total days in subfigures (**c**) and (**d**). We refer readers to Supplementary Table 1 where we explored linear mixed-effect models for fatigue and pain (complementing findings presented in subplots (**c**) and (**d**)).

The extent to which correlations within each participant (intra-person) between daily symptom and actigraphy measures varied is illustrated in Fig. 5. Actigraphy-assessed sleep duration the night following the daily PROM was not strongly correlated with self-reported pain or fatigue when using repeated measures correlations (see Supplementary Data 1), which is reflected by the wide range of intra-person correlation coefficients shown in Fig. 5a, b. In Fig. 5c, d, partial correlations (controlling for the remaining variables displayed in each plot) revealed a weakly negative association between actigraphy-assessed sleep duration (the night before the daily PROM) and self-reported fatigue, that was not present with pain. Similarly, mostly negative correlations were seen between actigraphy-assessed sleep regularity and self-reported fatigue. In contrast, a weak positive association between sleep duration (the night following the daily PROM) and self-reported fatigue was found in further analysis through linear mixed-effects models (see Supplementary Table 1). In Fig. 5a, b, PA (as shown indicatively by M10) also had mostly negative correlations with both pain and fatigue, but the association was strongest with self-reported fatigue when examining partial correlations (Fig. 5c, d) and in further analysis (see Supplementary Table 1), where the association between M10 and pain became weakly positive after controlling for fatigue and other actigraphy variables. Finally, a weak positive association was found between WASO (the night before the daily PROM) and self-reported pain, after controlling for other variables (see Fig. 5c and Supplementary Table 1). The extent to which these intra-person correlations varied based on hormonal and surgical status as well as sleep regularity is further detailed in Supplementary Figs. 7, 8.

### Participant-level associations between actigraphy and PROMs

**Fig. 6:**
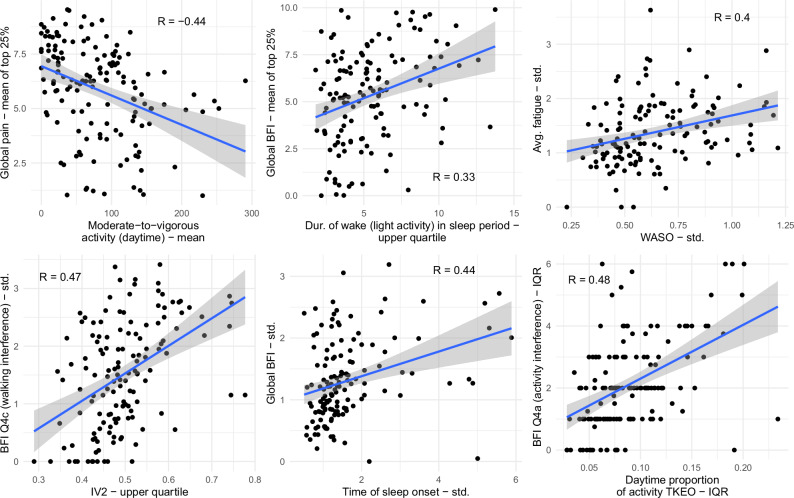
Scatter plots visually illustrating the relationship between indicative actigraphy measures (x-axis) and self-reports (y-axis) summarized across smartwatch cycles. A best-fit line is displayed in blue with a 95% confidence interval (shaded gray) along with the Spearman partial correlation coefficient (R), controlling for the smartwatch location (dominant vs. non-dominant wrist), age, and BMI. The displayed actigraphy measures were chosen to depict indicative strong correlations ( | R | >0.3), which also appeared robust with minimal outliers, involving different types of actigraphy measures (sleep vs. PA) with different types of summary measures (i.e., from different algorithmic families).

Comparing participant smartwatch cycles, using associations computed using summary measures over each smartwatch cycle or end-of-cycle questionnaire data, revealed numerous statistically strong Spearman correlations, and indicative examples of these correlations (i.e., strongest correlations that appeared robust, without outliers, from different broad algorithmic families) are shown in Fig. 6. Severe symptoms, as indicated by the mean of the upper 25% of global pain scores reported in a smartwatch cycle, were associated with lower mean moderate-to-vigorous physical activity (MVPA), and a strong correlation was also seen with mean global pain and mean upper 25% of global BFI scores (Fig. 6). Notably, actigraphy measures of PA summarized over smartwatch cycles (MVPA and moderate or vigorous activity) were more strongly correlated with summary measures of self-reported pain than fatigue, suggesting a relationship differing to that on a daily basis within participants. Sleep disturbance as indicated by the upper quartile of light activity during sleep was positively correlated with the mean of the upper 25% of global BFI scores (Fig. 6b).

Additionally, the variation in certain sleep and diurnal variability measures (Fig. 6c, d) were strongly correlated with variation in self-reported fatigue (either global BFI or individual BFI items), indicating that in general, smartwatch cycles with highly variable fatigue PROMs also displayed more variable sleep and activity rhythms. Furthermore, the variability of sleep duration across a smartwatch cycle was correlated with the EHP-30 pain subscale with a Spearman correlation of $R=0.3$, highlighting the statistical relationship between the variability of sleep patterns and self-reported symptom severity.

### Assessing changes following surgical intervention

**Fig. 7:**
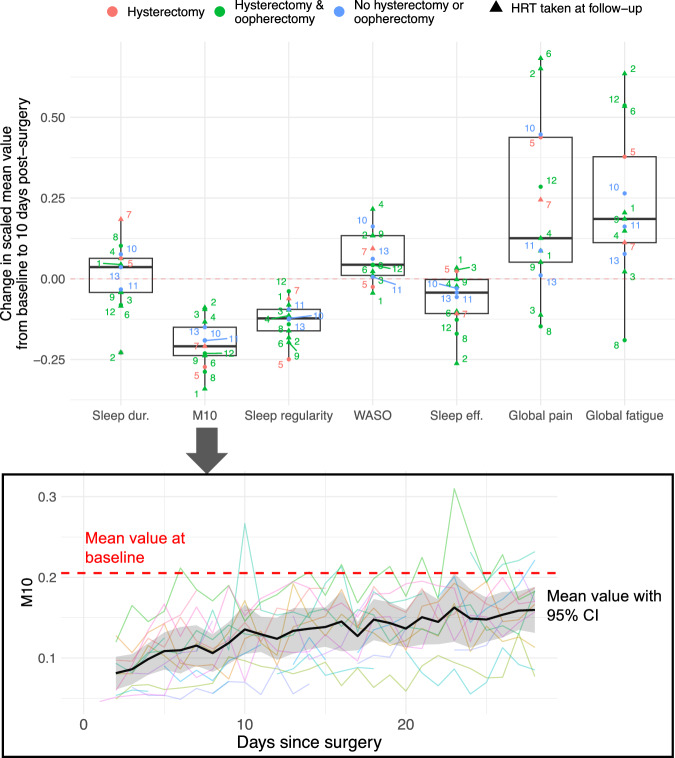
Changes in symptoms and actigraphy measures immediately following endometriosis surgery (including hysterectomy +/− oophorectomy) from participants in the surgical sub-study. The boxplots above show differences in min-max scaled actigraphy measures and self-report symptoms from baseline to the 10-days immediately post-surgery (top) for each of the labeled *n* = 13 participants (in random order). The line plot below shows of all *n* = 13 participant PA (as shown by M10) trajectories following surgery, with the mean value highlighted in black, the 95% confidence interval highlighted in gray, and the participant mean M10 baseline value shown by the red dotted line. For the mean and 95% CI, only days with data points from at least 50% of the participants were used. In the top figure, a minimum of three data points for a participant was available for the 10-day period, and min-max scaling across all daily data from all participants was applied.

We found that self-reported symptoms and actigraphy measures changed in the 10-day time-period following endometriosis surgical intervention (excision of endometriosis, with or without concurrent hysterectomy +/− bilateral salpingo-oophorectomy), as shown in Fig. 7. The decrease in actigraphy-assessed PA levels following surgery was consistent among the *N* = 13 participants with available data, which was followed by a gradual increase in PA levels for most participants. PA trajectories were generally the inverse of symptom trajectories, as pain and fatigue levels were elevated immediately following surgery followed by a gradual decrease. Increased sleep disturbance—as assessed using *wake after sleep onset* (WASO)—and decreased sleep efficiency was also seen in the actigraphy data for most participants in the 10-day post-operative window, and decreased sleep regularity was seen in all participants. In contrast, changes in actigraphy-assessed sleep duration in the post-operative period varied widely between participants, with six participants showing decreased sleep duration and seven with increased sleep duration.

**Fig. 8:**
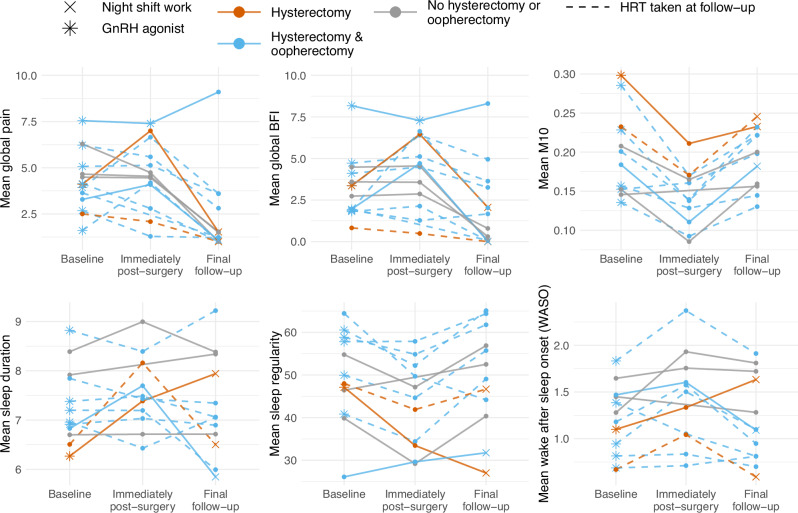
Line plots depicting changes in PROMs and actigraphy summary measures across smartwatch cycles for participants in the surgical sub-study. The line color depicts the type of surgery received (excision of endometriosis with conservation of uterus, or excision of endometriosis with concurrent hysterectomy±bilateral oophorectomy), the line type indicates whether HRT was taken at follow-up, while the cross (x) indicates where a participant reported working night shifts, and the asterisk (*) indicates where a participant received GnRH agonist during the smartwatch cycle. Indicative PA trajectories are shown using mean M10 values.

Summary values of actigraphy and PROMs from baseline to the 4-6 month follow-up are shown in Fig. 8. Improvements in self-reported pain and fatigue were the most consistent at follow-up (13 of 14 participants reported lower mean global pain scores compared to baseline, and 11 of 14 participants reported lower mean global BFI scores). Changes in actigraphy-assessed measures were more variable at follow-up, with five of 12 participants showing an increase in mean M10, 9 of 12 participants showing an increase in mean sleep regularity, and 5 of 12 participants showing a decrease in WASO.

## Discussion

We demonstrate the utility of wrist-worn actigraphy to offer new insights into objectively monitoring longitudinal endometriosis symptom trajectories and assessing endometriosis surgical intervention outcomes over and above what is offered by PROMs. Strong negative repeated-measure correlations ($R < -0.3$) were found between actigraphy-assessed PA measures and self-reported fatigue, suggesting that passively collected actigraphy offers potential towards tracking fatigue symptoms longitudinally. When comparing between smartwatch cycles from participants, strong correlations were found between PA and pain severity (${|R|} > 0.4$), between sleep disturbance and symptom severity (${|R|} > 0.3$), and between symptom variability and sleep and diurnal rhythm variability (${|R|} > 0.4$). These compelling findings provide the first objective evidence of associations between sleep, PA, and diurnal rhythms with endometriosis symptom severity which have previously only been explored using questionnaires. Finally, actigraphy assessment prior to and following endometriosis surgery (with optional concurrent hysterectomy and/or bilateral oophorectomy) revealed clear changes in PA, sleep disturbance, and sleep regularity immediately following surgery, which largely reflected concurrent symptom severity as assessed through PROMs.

Longitudinal actigraphy assessment of PA, sleep, and diurnal rhythms over multiple months has not previously been undertaken in a cohort of endometriosis patients, although one large-scale observational study has demonstrated the potential utility of tracking daily lifestyle habits such as exercise alongside symptoms in endometriosis^13^. We note that most actigraphy-focused studies in the research literature do not have repeated longitudinal data collection. By comparison, in this study we report on actigraphy from participants on different occasions throughout one year, providing up to 126 days’ worth of data per participant. In general, there has been a call for greater integration of digital technologies^21,22^, including in endometriosis research mirroring progress in other domains^6,12^. This study provides valuable insights into the utility and outcomes of taking this approach. In other chronic pain conditions, such as irritable bowel syndrome (IBS), migraine, and fibromyalgia, actigraphy has previously been used to identify sleep disturbances or PA levels compared to control populations, and in some cases to assess the temporal effects of sleep on symptoms (e.g., as a trigger for migraine)^6^. However, few studies have utilized longitudinal sensor-based assessment in endometriosis; one study evaluated associations with symptoms after laparoscopic surgery in a cohort of three participants^23^, and another followed patients after endometriosis surgery (among other participants) for one week to assess objective step counts^24^. In the former study, which used a non-contact sensor to detect sleep, strong positive correlations between the time from sleep onset to deep sleep and pain the following day were found in all three participants^23^. Another recent study used MVPA estimates and step counts from Fitbits over several weeks, finding that increased MVPA was associated with improved mental health, independent of pain, in a cohort of patients with chronic pelvic pain, many with endometriosis^25^. However, that study did not include assessment of fatigue, or any further measures of PA, sleep, and diurnal rhythms, as assessed in this study.

A key novel finding from our study is that actigraphy could provide important insights into endometriosis post-surgical outcomes. Consistent decreases in actigraphy-assessed PA (as indicated by M10) were seen among all participants in the period immediately following surgical intervention (with or without hysterectomy/oophorectomy). This corroborates findings presented in an earlier study using accelerometers to monitor PA after abdominal surgery, including laparoscopic hysterectomy (although participants with deep endometriosis were excluded in that study)^26^. In that study, following hysterectomy participants did not tend to reach their baseline PA levels by five weeks post-surgery. Although it is expected that PA typically decreases in the short-term following surgeries such as hysterectomy, the actigraphy data provide objective means and valuable insights into the actual rehabilitation trajectory.

In our study, increases in actigraphy-assessed sleep disturbance and decreases in sleep regularity were also present in almost all participants in the 10-day period following surgery; similar findings in a study of 16 women post-hysterectomy revealed a significant increase in WASO in the seven days following surgery compared to the seven days immediately prior to surgery^27^. That study, which included one participant with endometriosis among other diagnoses, also reported a significant increase in sleep duration following surgery, whereas in our study changes in sleep duration were more variable. A recent cross-sectional study using accelerometers and self-reported pain post-operatively, which included 60 participants following endometriosis surgery, did not find strong correlations between pain intensity and step count at 6-7 days post-surgery^24^. However, that cross-sectional approach is fundamentally different to our examination of associations between PA and symptoms *within* participants, which can provide more detailed insights into symptom trajectories.

Adherence to wearing smartwatches throughout our study was high, with a mean wear time of 87.3%, and was higher compared to PROMs adherence for most participants (80.5% mean adherence) as shown in Fig. 2c. Notably, the decrease in adherence within each smartwatch cycle was less abrupt for smartwatches compared to self-reports. However, acceptability responses and feedback from participants revealed several cases of discomfort from the smartwatches and suggestions to improve physical aspects of the watches (most commonly regarding discomfort from the watch strap, and in some cases the size of the device). Discomfort from the smartwatches and/or not wanting to wear the smartwatches were also provided as reasons for withdrawal from some participants, indicating that the devices themselves likely had some impact on smartwatch adherence levels. This suggests wear times could potentially be increased further by improving aspects of the smartwatches. Additionally, a common reason for withdrawing from the study was being “anxious/overwhelmed” (multiple choice) or similar (as indicated by free text), likely due to the burden of completing not only daily pain and fatigue ratings but also questionnaires at the end of each cycle. Thus, there are clear benefits to utilizing smartwatches that can passively collect information in long-term follow-up, thereby reducing the burden on participants and potentially improving adherence, as indicated by the increased adherence in the smartwatch data collection compared to daily PROMs shown in this study.

Comparing end-of-cycle EHP-30 outcomes to daily pain and fatigue scores suggested that retrospective reports of symptoms may in fact tend to reflect the worst symptoms experienced, which is a common occurrence reported within the wider literature on PROMs^14^. In particular, past research on self-reporting of pain intensity has similarly found that retrospective reports of total pain tend to be strongly associated with the maximum pain intensity experienced^28^. As no other studies have evaluated adherence to wearing smartwatches in endometriosis to our knowledge, no direct comparison for smartwatch adherence is available. For daily PROMs, previous studies in a clinical trial setting have indicated high adherence, such as validation of a daily endometriosis diary, which had 90% adherence over one menstrual cycle^29^. However, adherence to completing PROMs is dependent on the length and format of self-reports, cohorts, reminders given, and duration of the reporting period^14,30^.

When examining correlations between daily actigraphy measures and daily PROMs within each participant, strong negative correlations between actigraphy-assessed PA (as measured by M10) and fatigue suggest the potential utility of using smartwatches to remotely monitor fatigue state. In contrast, weak and varying associations were found between sleep measures and symptom severity, potentially due to the nature of actigraphy-assessed sleep, which relies on detecting periods of sustained inactivity and can differ from gold-standard sleep assessment. We remark that although no repeated measures correlations stronger than ${\rm{|R|}} > 0.4$ were identified, empirical work suggests that in clinical applications correlations with ${\rm{|R|}} > 0.3$ are typically considered statistically strong^31^, and may be important for clinical interpretation or in the context of monitoring using multivariable models^32,33^. This follows empirical statistical principles, where the presence of statistically strong relationships indicates that there is high probability of building accurate statistical learning models using explanatory variables (e.g. the actigraphy-based measures) towards estimating the outcome (e.g. self-reported pain and fatigue)^32^. As illustrated in Supplementary Fig. 2, two participants demonstrated frequent abnormally long detected sleep duration (even up to 20 h), which very likely reflects prolonged sedentary activity rather than actual sleep (see also Supplementary Fig. 9). Upon visualization of the underlying raw actigraphy data, the long periods of inactivity were not easily distinguishable from potential sleep periods, and thus were retained as valid readings of ‘sleep’ although it is likely the participants were not sleeping for the entire duration. Therefore, resulting associations between ‘sleep duration’ and self-reported symptoms may in fact reflect sustained inactivity in certain cases, complicating the varying intra-person correlations illustrated in Fig. 5.

Despite this potential conflation between sleep and inactivity, the partial correlations between actigraphy measures and fatigue, which controlled for other actigraphy measures and pain (see Fig. 5c, d), revealed a weak negative association between sleep duration the previous night and fatigue the following day. However, this was not statistically significant in the mixed effects models (see Supplementary Table 1), suggesting lack of sleep may increase fatigue but is likely not the main factor. Additionally, the tendency to recuperate sleep after a night with reduced sleep duration can also obscure the effect of symptoms on sleep and vice versa; the strong correlations found between variability in sleep duration and variability in symptom severity (Fig. 6) support this hypothesis.

Overall, the negative daily correlations between PA (M10) and fatigue were strongest and consistent across participants (up to $R=-0.35$), suggesting that although sleep could have a potential influence on symptoms the following day, low levels of PA are much more indicative of high fatigue levels than sleep. However, it is worth noting that several participants demonstrated weak or positive intra-person correlations between M10 and fatigue, and partial correlations indicated a slightly positive association between M10 and pain after controlling for fatigue, suggesting it may be of benefit to consider associations on an individual basis, as PA could also potentially increase fatigue levels. Furthermore, associations between pain and actigraphy-assessed PA measures were notably weaker when compared to fatigue, and daily pain scores were not strongly correlated ( | R | > 0.3) with any daily actigraphy measures (see Supplementary Data 1). After controlling for fatigue and other actigraphy measures, further examination through mixed-effects models revealed a positive association between sleep disturbance (WASO) the previous night and pain (statistically significant as shown in Supplementary Table 1).

Actigraphy-assessed sleep regularity was also negatively associated with daily self-reported fatigue (Fig. 5), even after controlling for pain and other actigraphy variables (see Fig. 5c and Supplementary Table 1), indicating that with more severe symptoms, sleep patterns tended to be less aligned. However, sleep regularity—as the computation is based on periods of detected sustained inactivity (not only within the sleep period)—was also weakly correlated with M10 (repeated measures correlation of 0.23)—and thus associations may be influenced by long periods of sustained inactivity during the day for certain participants. Furthermore, as explored in Supplementary Figs. 7, 8, the hormonal and surgical status or sleep patterns could potentially impact the strength of intra-person correlations, likely due to the symptom severity and variability as well as the presence of cyclical vs. non-cyclical symptoms.

When summarizing actigraphy measures and symptoms over smartwatch cycles (see Fig. 6), surprisingly, mean self-reported fatigue was much less strongly correlated with actigraphy-assessed PA levels than mean self-reported pain, suggesting that pain symptoms may be impacting overall PA levels more than fatigue, while within-person changes in PA may be more reflective of fatigue level. Actigraphy-assessed PA has similarly been found to be inversely associated with symptom severity in a study of chronic pain (women with fibromyalgia)^34^. Furthermore, smartwatch cycles with higher extremes of sleep disturbance also tended to have higher extremes of symptom severity (Fig. 6), which is in line with previous questionnaire-based studies of endometriosis, where patients with more severe pain symptoms reported greater fatigue and greater sleep disturbance compared to those with minimal pain^35^.

We acknowledge there are some limitations in the study. The relatively small number of participants suggests that findings should be interpreted tentatively. Similarly, although a limited number of participants underwent surgery, we demonstrated that several changes in actigraphy measures were consistent across all (or almost all) of the participants immediately after surgery. Thus, we are reasonably confident the presented findings would very likely generalize. Another limitation of this study is the representativeness of the cohort: data was collected from a single center with participants from a similar background and an over-representation of participants with deep or ovarian endometriosis compared to their prevalence within the general population. Furthermore, we acknowledge that the lack of a control group in this study precludes any direct comparison of absolute measures of PA or sleep to a healthy population. It is also possible that the changes in actigraphy measures immediately following surgery could be similar in other surgical cohorts. However, as this study aimed to assess associations between actigraphy and PROMs in endometriosis, examining daily associations would not be suitable in a population with limited or no symptom severity. Finally, as previously discussed, the actigraphy-assessed sleep duration was likely overestimated for certain participants in the cohort who demonstrated highly sedentary behavior, often during periods of severe pain and fatigue.

Future work could involve larger studies, potentially with more frequent PROMs, to understand the complexities of the relationship between pain and fatigue symptoms and lifestyle behaviors such as sleep and PA. The impact of pain medication and other lifestyle factors could also be considered in future studies. Additionally, more participants with different surgical interventions could provide a more robust analysis of objective changes at a six-month follow-up. Qualitative and feasibility studies could also provide more insight into how features of wearable devices, including different types of wearable devices, could improve adherence in longitudinal studies. Finally, in further work we plan to assess the utility of the daily actigraphy measures for remote monitoring of symptom severity using individualized machine learning approaches. Individualized modeling approaches, or more complex non-linear models that better account for the time-series leading up to the symptom report, could potentially improve prediction of pain, whereas in this exploratory study only weak associations were found between daily actigraphy measures and pain.

Given that endometriosis is a chronic condition, it is imperative to collect longitudinal data to understand symptom trajectories and assess interventions. These interventions are typically assessed through sparse PROMs, which are known to be subject to recall bias when not regularly collected, and burdensome to participants to collect longitudinally. In this study, we found clear utility in using passive data collection through smartwatches, including high adherence, the potential for remote monitoring of symptoms such as fatigue, and insights into heterogeneous symptom severity trajectories, particularly post-operatively. In summary, this is the first study in endometriosis to report how objective longitudinal assessment of PA, sleep, and diurnal rhythms through wrist-worn wearable devices can provide insights into daily symptoms over and above PROMs. We envisage this work will contribute towards establishing a clinically useful pathway to facilitate individualized objective insights into endometriosis symptom trajectories, which could be embedded in future interventional or large-scale digital phenotyping studies.

## Methods

### Study design

**Fig. 9:**
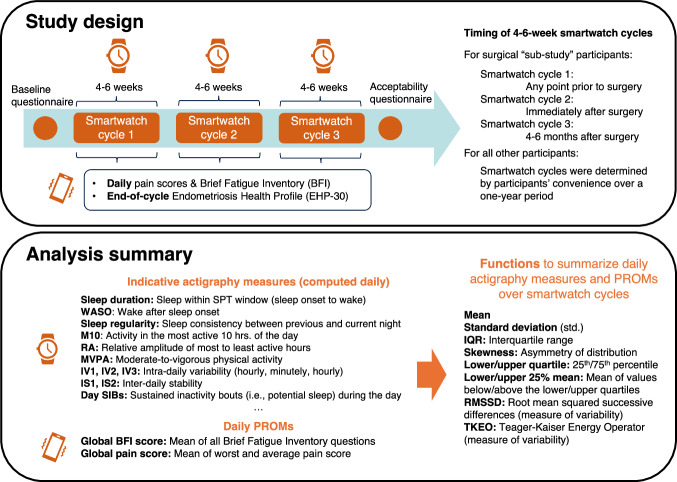
Diagram of study design and analysis summary. Included in the analysis summary is a glossary of indicative actigraphy measures and daily PROMs used in the analysis. For definitions of all daily actigraphy measures used in the analysis see Supplementary Data 1 (under the tab “Variable Definitions”).

Participants were recruited from gynecology out-patient departments and endometriosis service, including those on waiting lists for surgery. Inclusion criteria were defined as follows: being aged 16 or over, a diagnosis of endometriosis on imaging (for deep or ovarian endometriosis only) or at previous laparoscopy (all subtypes), no malignancy, and not currently pregnant. The study design is illustrated in Fig. 9. Participants took part in the study for up to three 4-6-week periods, which herein are referred to as *smartwatch cycles* (the term is used only to refer to cycles of wearing the smartwatch, not menstrual cycles). Smartwatch cycles could be completed whenever convenient, generally over a maximum period of 12 months. Participants were contacted by telephone by research nurses to schedule subsequent smartwatch cycles. A subset of patients who had previously been diagnosed with deep endometriosis and who planned to receive surgery to excise the endometriosis, with or without additional total hysterectomy±BSO, were recruited to the surgical “sub-study”, where they were asked to complete the first smartwatch cycle at any point prior to surgery, the second immediately following surgery, and the third approximately 4-6 months following surgery. Other participants could potentially have surgery during the study, but only those recruited to this “sub-study” and with deep or ovarian endometriosis found at surgery were required to complete the smartwatch cycles on this schedule and thus could be compared in further analysis. Consistent timing of smartwatch cycles was not required for all other participants for several reasons: to encourage ongoing participation by allowing hospital visits at intervals convenient for participants, and because no time-wise comparisons were made aside from the surgical sub-study (i.e., first smartwatch cycles were not compared directly to other first smartwatch cycles, etc.).

During each smartwatch cycle, participants were solicited to complete daily self-reports (PROMs) of symptoms and were asked to wear the GENEActiv smartwatch. In the final week of each smartwatch cycle, participants were also asked to complete QoL questionnaires. At baseline and following each smartwatch cycle, details of any medication changes, surgery, menstruation, holiday, and other relevant information was collected by research nurses. At the end of the third smartwatch cycle or at withdrawal, participants were also asked to complete an acceptability questionnaire. All self-reported data, medical history, and surgical data was collected using REDCap electronic data capture tools hosted at the University of Edinburgh^36,37^.

### Patient-reported outcome measures (PROMs)

Participants submitted daily pain and fatigue scores using (i) two questions assessing “average pain today” and “worst pain today” on a linear 10-point numeric scale from 1 (“no pain”) to 10 (“worst pain imaginable”), and (ii) the BFI. The BFI consists of nine items rated on an 11-point numeric scale, where the first three items ask to rate fatigue “right now”, “usual level of fatigue during the past 24 hours”, and “worst level of fatigue during the past 24 hours”^38^. The following six items assessed how, over the past 24 h, fatigue interfered with the following: (1) general activity, (2) mood, (3) walking ability, (4) normal work, (5) relations with other people, and (6) enjoyment of life. Daily PROMs could be completed through a daily SMS, or email link, or on paper, for inclusivity and adapting to participants’ preferences.

Information on demographics (ethnicity, education), medication use, and medical history, was collected by research nurses at baseline. After each smartwatch cycle, participants additionally reported any issues wearing the smartwatch, holidays, dates of menstruation, shift work, pregnancy, hormonal and pain medication taken, and any surgery or hospital visits.

Participants completed the EHP-30 questionnaire at baseline and in the final week of each smartwatch cycle. The EHP-30 is a clinically validated questionnaire to assess the impact of endometriosis on health-related quality of life (HRQoL), consisting of 30 questions each on a Likert scale from 0 (Never) to 4 (Always). The 30 questions are categorized into five subdomains related to “pain”, “control and powerlessness”, “social support”, “emotional wellbeing”, and “self-image”^39^. The questionnaire requires participants report on their symptoms *retrospectively*, “during the last 4 weeks”^39^. Other questionnaires were also completed along with the EHP-30 but were not analyzed in this study.

### Smartwatch data

Consented participants were asked to wear the GENEActiv Original smartwatch (https://activinsights.com/) for the duration of each smartwatch cycle. The GENEActiv watches were configured to collect tri-axial acceleration data at 10 Hz (which allows for a data collection period of at least 30 days on a single charge), which we have shown in previous work is fully sufficient for day-to-day PA and sleep assessments^17^. Collecting data *longitudinally* (in the actigraphy setting this is often taken to refer to > 2-3 weeks) is particularly useful to quantify diurnal rhythms (see below for details): many actigraphy studies are limited to only collecting data for a single week, which severely limits the extent of the information that can be extracted. The watches captured a dynamic range of ±8 g, where g are the units of acceleration (equal to acceleration due to gravity) with a resolution of 12 bit (3.9 mg). In addition, the watches incorporate a sensor for ambient light on a range of 0–3000 lux with resolution of 5 lux, and a temperature sensor on a range of 0-60 degrees Celsius with resolution of 0.25 degrees.

#### Adherence analysis

To compute the average adherence of participants for both smartwatches and daily PROMs, smartwatch cycles with valid smartwatch data or with more than one recorded daily PROM were included. Thus, adherence for participants that dropped out after the first smartwatch cycle, for instance, would only be computed for that first smartwatch cycle. To account for the bias due to participants that did not participate in all three smartwatch cycles, we also compared adherence between the dropout and non-dropout groups (i.e., also computing adherence per smartwatch cycle for those participants that completed all three smartwatch cycles).

### Processing of actigraphy data

To process the raw actigraphy data we used the R-package GGIR (version 3.0.6, see Supplementary Table 2 for the exact configuration parameters used). GGIR provides open-access tools for device calibration, non-wear and sleep detection, and extraction of a large range of sleep and PA measures^40^. Additionally, we processed the raw data using a further open-source approach with the MATLAB Actigraphy Toolbox developed in house and then implemented in R, which we have reported on in previous work^41^.

Raw actigraphy data must first be calibrated prior to any further processing, as each individual tri-axial accelerometer has device-specific offsets and therefore outputs must be aligned. For data processed using GGIR, the autocalibration process built into the package was used as detailed in van Hees et al.^42^, relying on periods of non-wear to take local gravity into account. For data processed independently of GGIR, outputs were calibrated using the offsets stored in the device by the manufacturer using the device-specific GENEARead package (version 2.0.10).

To detect periods where the device was not worn (non-wear), GGIR utilizes rolling 15-min intervals centered (centered in a 60-min interval to take into account the periods before and after) and identifies acceleration below a specific threshold within that interval^43^. However, we found frequent misdetection of non-wear using this method, and thus we also adapted an established non-wear detection method that incorporates the temperature sensor readings, which can provide clear indication of when the device is worn^44^. For the endometriosis participants in this study, classification of non-wear periods indicated that adapted temperature thresholds were needed. To detect periods of non-wear periods in the actigraphy data, we adapted a previous non-wear detection algorithm by Zhou et al. that utilises the temperature sensor incorporated into the GENEActiv watches in addition to acceleration data^44^. The Zhou et al. method uses a threshold based on the standard deviation of acceleration values as well as a temperature threshold within a rolling window. Here, we first computed rolling averages of temperature across 5-minute windows (T_smooth_), and then detected periods of at least 90 minutes where T_smooth_ was lower than a chosen threshold T_0_ or the change in T_smooth_ from the previous minute was lower than -0.5 °C. Of those periods, those where the *rate of change acceleration movement* (ROCAM) (see below, paragraph on acceleration summary measures) was below 0.025 were set as non-wear, and if two non-wear periods had less than 15 min in between, this period was also designated as non-wear. T_0_ was chosen as either 26 degrees Celsius or the 5^th^ percentile of temperature readings, whichever was larger.

Detecting non-wear periods of at least 90 min was first performed for a more accurate assessment of the average temperature when the smartwatch was worn. Subsequently, to detect non-wear periods between 15 and 90 min, a new temperature threshold T_1_ was then chosen as one standard deviation below the mean temperature (up to a maximum temperature of 24 degrees Celsius) after excluding periods already designated as non-wear. If the 5^th^ percentile of all temperature readings was larger than T_1_, then this temperature was used instead, and if the smartwatch was worn for fewer than 3 full days after the first iteration, T_1_ was set to 24 degrees Celsius. The same thresholding approach was used as described for long non-wear periods but followed by a filtering method where only short non-wear periods were kept, where the first 5 minutes of the interval was at least 2 degrees higher than the final 5 minutes, as within shorter non-wear periods it is typical to see a sudden drop in temperature. This non-wear detection algorithm was found to be accurate for the cohort in our study, as each participant’s data was visually assessed following non-wear classification.

The sleep detection method incorporated into GGIR was primarily used in this study, as described in ref. ^45^, which detects periods of time when changes in the arm angle are below a threshold of 5 degrees over at least 5 minutes (“sustained inactivity”). As no self-reported sleep logs were collected in this study, further processing was used to detect the most likely sleep period time (SPT) window using a heuristic algorithm detailed in van Hees et al.^46^. Subsequently, periods of wake during the sleep period (WASO) were computed as periods not detected as sustained inactivity within the SPT-window. However, many sleep detection methods, including that by van Hees et al.^45,46^, were designed to work well primarily in healthy individuals, and although some work has been undertaken to explore accuracy in cohorts with sleep disorders^47^ (e.g., van Hees et al.^45^ validated their method against polysomnography from cohorts with sleep disorders), it is unknown how well these algorithms perform in free-living contexts for individuals with various conditions that may affect sleep such as in endometriosis. To provide a comparator to GGIR-based sleep detection, we also used the sleep detection method presented in Tsanas et al.^41^ that was developed and validated both for healthy controls and people with post-traumatic stress disorder where the hallmark symptoms include frequent sleep disturbances. The method by Tsanas et al. utilizes thresholds based around measures of acceleration and ambient light collected by the devices, and crucially can allow for the detection of multiple sleep periods which is relevant for cohorts with severely disturbed sleep. We remark that although that sleep detection algorithm was not validated against polysomnography data, it was demonstrated to match well self-reported sleep onset and offset times in participants’ sleep diaries.

After observation of sleep detection results in specific participants in this study cohort, we also decided to detect periods of ‘low variation’ using a similar method to sleep detection by Tsanas et al., with low variation defined using the average of successive differences of each axis (the threshold was chosen using visualization of nights of sleep). Measures relating to low-variation periods, such as overall duration and percentage duration within the SPT period, were subsequently extracted.

Similarly to sleep detection, to extract daily measures of PA and diurnal rhythms, we utilized both GGIR and additional approaches as presented in Tsanas et al. ^41^. A typical pre-processing step towards characterizing actigraphy data is using an *acceleration summary measure* to project the three-dimensional acceleration data onto a vector. Van Hees et al. used the Euclidean Norm Minus One (ENMO) acceleration summary measure (with negative values rounded to zero, also referred to as ENMONZ) for the actigraphy measures computed using GGIR^48^, whereas all additional measures utilized the recently proposed ROCAM, which has been shown to outperform alternative widely used acceleration summary measures in terms of mapping onto PA levels and sleep^17^. Using the resulting vectors from the application of the acceleration summary measures, we subsequently computed actigraphy measures to characterize the magnitude and patterns of movement per day, for instance the most active 10 h (M10) or least active five hours (L5) of the day, or the relative amplitude of most and least active hours (RA). Additionally, using GGIR we extracted PA measures such as inactivity and light, moderate, or vigorous PA, as well as moderate-to-vigorous PA (MVPA). PA intensity measures were extracted using default cut-points for the GGIR algorithm. Although these cut-points were similar to those established by previous studies using GENEActiv devices on the non-dominant wrist^49^, participants in our study were allowed to choose the wrist placement of the smartwatch. Although these differences could introduce bias to comparisons between participants, it would not generally influence the findings within participants (only two participants recorded changing wrists partway through the study). A full list of extracted actigraphy measures using both GGIR and other approaches is detailed in Supplementary Data 1 (we clarify that the source indicated therein refers to the implementation we used in this study rather than reflecting where each actigraphy measure was first proposed).

Additionally, to quantify sleep and circadian rhythms, we utilized a measure of sleep regularity, the sleep regularity index (SRI), proposed by Philipps et al.^50^ and modified to apply to day-pairs as implemented in GGIR. The SRI measure compares the sleep state within 30-s time-points 24 h apart (e.g., day *k-1* and day *k* as applied for day-pairs as in GGIR). The resulting value ranges from -100 to 100, with 100 representing perfectly aligned sleep periods^50,51^. Lastly, the temperature sensors incorporated into the devices used were also used to extract further daily measures relating to diurnal rhythms, as proposed in our work previously^19^. Daily actigraphy measures were ignored if the smartwatch was worn for less than 75% of the 24-h period either starting at 12am (for measures assessing daytime sleep/PA or across the full 24 h) or at 12 pm (for sleep measures). Sleep regularity values were also only included if the GGIR-assessed validity (using non-wear detection) was greater than 80%.

### Processing of PROMs

For the daily PROMs reporting pain (average and worst) and fatigue (BFI), both the individual question scores and global scores were used. To compute the global scores, we used the mean of the two pain scores (*global pain*), and similarly for the BFI we took the mean of the scores on the nine questions (*global fatigue*)^38^. For the EHP-30 questionnaire, which was evaluated near/at the end of each smartwatch cycle, we examined the scores from the subdomains (*pain_ehp30, control_ehp30, emotion_ehp30, social_ehp30, self_ehp30*) in addition to the total of all 30 questions (*ehp30_overall)*^52^ after normalizing to a scale of 0 to 100.

The PROMs in this study were collected using REDCap and the collected data was processed in R after being exported. Ad-hoc corrections to the raw data (e.g., where PROM dates were clearly mistakenly entered) were made prior to any further processing. Daily self-reports completed between 5 pm on the associated date and 5 am the following morning were considered within the “correct” time-window for the purposes of de-duplicating entries, where if duplicate entries were present, only the entry within the correct timeframe, or if unavailable then within a “feasible” timeframe (anytime on the associated day or following day) was included.

### Summarizing daily actigraphy measures and PROMs

To compare the daily PROMs and daily actigraphy measures with end-of-cycle questionnaires and participant-level data (e.g., BMI, age, diagnosis), we computed summary measures of the day-level data across each smartwatch cycle and across individuals. To limit bias from participants or smartwatch cycles with large amounts of missing data, when computing measures across participants, we included only participants with at least 10 non-missing values to compute the mean, and at least 20 values to compute all other summary measures, where “missing” means either no PROM was submitted for that day or the 24-h wear-time was below 75%. Similarly, when computing across smartwatch cycles, we included only smartwatch cycles with fewer than 10 non-missing values to compute the mean, and 20 values to compute all other summary measures.

We then extracted the mean value, standard deviation, skewness, interquartile range (IQR), the upper quartile (75^th^ percentile) and lower quartile (25^th^ percentile) using actigraphy data only, and the mean of upper and lower quartile values (i.e., highest and lowest 25% of daily values, respectively) using both self-report and actigraphy data. For PROMs, the mean of the upper and lower quartile values was used to better summarize the ordinal scales with bounded and discrete values (e.g., with only 10 values).

To compute further variability measures across smartwatch cycles, we first imputed any remaining missing values using linear interpolation (through the *imputeTS* package in R) with a maximum gap of three missing values, such that if more than 3 consecutive values were missing, they would not be imputed. This imputation was performed before computing variability measures that utilize consecutive daily values, as missing data may introduce unrealistic ‘jumps’ between values that are falsely treated as consecutive days. On the imputed data (with remaining missing values removed), we then computed the Teager-Kaiser energy operator (TKEO) and and root mean squared successive differences (RMSSD), as have been used in other studies^53^, which are defined in Eq. (1) and Eq. (2), respectively:

1 $${TKEO}=\frac{1}{N}\sum _{i=2}^{N-1}({{x}_{i}}^{2}-{x}_{i-1}{x}_{i+1})$$

2 $${RMSSD}=\sqrt{\frac{1}{N}\sum _{i=1}^{N-1}{({x}_{i+1}-{x}_{i})}^{2}}$$

### Statistical associations

Repeated measures correlations were used to examine pairwise associations between daily self-reports and the daily actigraphy measures, using R-package *rmcorr*^54^ (the package also computes *p*-values using the F-ratio). This approach allowed for capturing only the within-subject variation rather than between-subject variation. Statistical associations were interpreted as “statistically significant” at a threshold of *p* < *0.05*. Intra-person correlations were defined by computing Pearson correlations using each individual participant’s data separately. Partial correlations were also computed using Pearson or Spearman correlations using the R-package *ppcor*^55^. Partial correlations were used to control for the effect of other variables (e.g., possible confounding factors) when examining the association between two variables. To fully investigate associations between daily actigraphy variables and daily PROMs, we used linear mixed-effects models to further account for potential autocorrelation within a participant’s repeated measures (given that measures on days close to each other may be more strongly correlated). Specifically, we modeled the daily PROMs (“fatigue” is the global BFI score, and “pain” is the global pain score) according to Eq. (3) and Eq. (4):

3 $${{Fatigue}}_{i,j}={\beta }_{0}+{\beta }_{1}\cdot {{pain}}_{i,j}+{\beta }_{2}\cdot {M10}_{i,j}+{\beta }_{3}\cdot {M10}_{i-1,j}+{\beta }_{4}\cdot {{SleepDur}}_{i,j}+{\beta }_{5}\cdot {{SleepDur}}_{i-1,j}+{\beta }_{6}\cdot {{WASO}}_{i,j}+{\beta }_{7}\cdot {{WASO}}_{i-1,j}+{\beta }_{8}\cdot {{SleepReg}}_{i,j}+{u}_{j}+{\varepsilon }_{i,j},$$

4 $${{Pain}}_{i,j}={\beta }_{0}+{\beta }_{1}\cdot {{fatigue}}_{i,j}+{\beta }_{2}\cdot {M10}_{i,j}+{\beta }_{3}\cdot {M10}_{i-1,j}+{\beta }_{4}\cdot {{SleepDur}}_{i,j}+{\beta }_{5}\cdot {{SleepDur}}_{i-1,j}+{\beta }_{6}\cdot {{WASO}}_{i,j}+{\beta }_{7}\cdot {{WASO}}_{i-1,j}+{\beta }_{8}\cdot {{SleepReg}}_{i,j}+{u}_{j}+{\varepsilon }_{i,j},$$

where ${{Fatigue}}_{i,j}$ represents the global BFI score on day *i* from participant *j*, the $\beta$ coefficients represent the fixed effects, ${u}_{j}$ represents the random effect of participant *j*, and ${\varepsilon }_{i,j}$ represents the residual error. Similarly, ${{Pain}}_{i,j}$ refers to the global pain score on day *i* from participant *j*. Additionally, we imposed an autoregressive process (order 1) as the correlation structure between residual errors for repeated measures from an individual participant, where distance between two errors was determined by the day of enrollment in the study (such that adjacent repeated measures are considered more strongly correlated). We refer to Gałecki et al.^56^ for further clarification of linear mixed-effects models with specific correlation structures. The models were implemented in R using package *nlme* (version 3.1-166). Only a small indicative set of actigraphy variables was chosen to avoid collinearity and illustrate associations between the main constructs: sleep, physical activity, and diurnal rhythms. All variables were standardized prior to model fitting.

When computing associations, days where any of the variables of interest (i.e., two variables in pairwise associations and all relevant variables for partial correlations and linear mixed-effects models) were missing were excluded. Spearman correlations were used to examine pairwise associations between measures summarized at a smartwatch cycle or participant level, and thus all correlation coefficients presented throughout as ‘*R*’ refer to the Spearman coefficient unless indicated otherwise. Correlations were regarded as *statistically strong* when ${|R|} > 0.3$, which is common in clinical applications^31^. Comparisons between subgroups, such as by treatment received, were visually examined using boxplots or distribution plots; due to the limited sample size and exploratory nature of the study, these visualization approaches were used to provide an overview of potential group differences (including outliers) which should be tentatively interpreted.

As an exploratory study assessing statistical associations with a large number of actigraphy measures (many of which were highly correlated) with no pre-specified analysis, no adjustment for multiple comparisons was applied as this would likely result in overlooking many potential key statistical associations. Thus, in this study we primarily focused on the statistical strength of associations (rather than statistical significance) as an indication of potential relationships that should be interpreted tentatively. Assumptions relating to the independence of samples were addressed by using methods that account for repeated measures, and non-parametric alternatives (Spearman correlation coefficient) were used where appropriate. Although Pearson correlations were used to compute intra-person correlations, only point estimates were used and only computed where enough valid entries ( > 20) were present; these correlations were used to illustrate variation in the data that was further investigated in mixed-effects models that accounted for autocorrelation in repeated measures.

### Ethical approval

The data in this study was collected as part of the EndoTECH study ‘Understanding the symptoms of endometriosis using Smartwatch technology’ performed in NHS Lothian, Scotland, UK, and was approved by the relevant research ethics committee (West of Scotland REC 5, ref. 21/WS/0092). Informed consent to participate in the study were obtained from all participants. The research has been performed in accordance with the Declaration of Helsinki.

## Supplementary information

Supplementary Information

Supplementary Data 1

## Data Availability

The patient data from this study are not available from public data repositories. Non-identified data may be shared on reasonable request for use in studies investigating women’s health conditions, subject to ethical approval. Requests for data sharing should be made to Andrew.Horne@ed.ac.uk.

## Code availability

All code was developed using R (version 4.4.0). We employed common R libraries for processing, analysis, and visualisation (e.g., *data.table, ggplot2, plyr, dplyr)* as well as packages specific for actigraphy processing and time-series analysis as specified in the Methods section. Code for the pre-processing of the raw actigraphy data, extraction of diurnal rhythm measures, pre-processing of PROMs, and the full analysis can be found at https://github.com/katherineedgley/endometriosis-symptom-tracking. The code for sleep detection as adapted from Tsanas et al.^41^ can be made available to bona fide researchers upon reasonable request and subject to license permitting free use for academic research.
