# Supplementary material for: Insights into endometriosis symptom trajectories and assessment of surgical intervention outcomes using longitudinal actigraphy
Source: NPJ Digit Med. 2025 May 2;8:236. doi: 10.1038/s41746-025-01629-8 (PMC12048534; doi:10.1038/s41746-025-01629-8)
Supplement: Supplementary file 1 — Supplementary Information [file 41746_2025_1629_MOESM1_ESM.pdf]

Supplementary Information

Table of contents

Supplementary Data 1. Spreadsheet of repeated measures correlations, associated *p*-values, and definitions of all daily actigraphy measures used in the study
Supplementary Note 1. Adherence: Inclusion and descriptive statistics
Supplementary Note 2. Exploration of associations with demographics and possible confounders
Supplementary Figure 1. Heatmap of hormonal medication and comorbidities
Supplementary Figure 2. Sleep duration and MVPA duration daily values by participant
Supplementary Figure 3. Symptom trajectory for indicative participant after beginning GnRH agonist
Supplementary Figure 4. Investigation of potential confounders and associations with demographic variables
Supplementary Figure 5. Scatterplots and boxplots of cycle-level mean and standard deviation in global pain and fatigue by type of hormones taken and surgical treatment
Supplementary Figure 6. Boxplots of indicative actigraphy summary measures across cycles by hormones taken or surgical status
Supplementary Figure 7. Intra-cycle correlations by hormonal medication or surgical status
Supplementary Figure 8. Intra-person correlations compared to mean level of sleep regularity
Supplementary Figure 9. Indicative example from participant P54 of variable sleep patterns and low physical activity together with severe daily symptoms
Supplementary Table 1. Results of linear mixed-effects models predicting daily global BFI scores and global pain scores using set of actigraphy measures
Supplementary Table 2. GGIR configuration parameters.

Supplementary Data 1.
Spreadsheet of repeated measures correlations, associated p-values, and definitions of all daily actigraphy measures used in the study.

The Supplementary Data 1 file contains all repeated-measures correlations between actigraphy measures and PROMs ("Repeated-measures correlations" tab) with associated p-values ("P-values" tab) and descriptions of all daily actigraphy measures ("Variable definitions" tab). Repeated measures correlations were computed using all available data from $n = 66$ participants, with a minimum of 3,589 and maximum of 4,521 degrees of freedom.

Supplementary Note 1.
Adherence: Inclusion and descriptive statistics.

The duration of each smartwatch cycle was initially set as six weeks, but this was reduced to four weeks partway through the study due to the smartwatch battery lifespan, resulting in the first $n=42$ participants completing six-week smartwatch cycles and the remaining $n=26$ participants completing four-week smartwatch cycles.

Of the $n=68$ participants that consented to the study, $n=66$ participants returned at least one smartwatch ($n=11$ participants had one 4-6-week cycle with at least one day of valid smartwatch data, $n=7$ participants had two cycles, and $n=48$ participants had three cycles). Due to smartwatch error, three smartwatch cycles (N=3) were not captured and $N=1$ smartwatch cycle was only partially captured. Two smartwatch cycles ($N=2$) were missing due to smartwatches not being returned. All but one participant ($n=67$) completed at least one daily PROM: $n=10$ participants submitted PROMs for only one smartwatch cycle, $n=5$ for two smartwatch cycles, and $n=52$ for all three smartwatch cycles.

When examining the first 28 days of each smartwatch cycle, the mean percentage of smartwatch wear time (where smartwatch cycles that were missing due to not being returned or partially/fully missing due to smartwatch error were ignored) across all $n=66$ participants with available data was 87.3% (SD=19.5%). When excluding participants that withdrew before three smartwatch cycles, this rose to 92.2% (SD=12.4%). Of the $n=67$ participants with more than one daily PROM, the mean adherence to PROMs was 80.5% (SD=20.9%), which rose to 87.6% (SD=10.7%) when excluding participants that withdrew before three smartwatch cycles.

Supplementary Note 2.
Exploration of associations with demographics and possible confounders

To investigate potential factors that could confound the relationship between symptoms and actigraphy measures, we also compared baseline characteristics, such as BMI and age, as well as where the smartwatch was worn (dominant or non-dominant wrist), with physical activity and sleep. Notably, participants with a diagnosis of deep or ovarian endometriosis subtypes (with or without superficial peritoneal disease) were older, with a mean age of 36.8 (SD=6.6) among participants with either deep or ovarian disease, compared to 29.0 (SD=6.1) among participants with only superficial peritoneal disease. When comparing between participants, i.e., where symptom and actigraphy measures were summarized across all available smartwatch cycles from each participant, we found that BMI was strongly correlated with a range of physical activity measures (e.g., acceleration during 'inactive' periods was strongly correlated with BMI with $R = -0.53$). Surprisingly, BMI was also strongly correlated with temperature measures, such as the mean of the maximum temperature during sleep ($R = -0.56$), as well as minimum and mean temperature during sleep. However, no statistically strong ($|R| > 0.3$) correlations were found between BMI and PROMs.

The age of participants, on the other hand, was negatively correlated with multiple PROMs, including the total EHP-30 score ($R = -0.34$), which could suggest that older participants have tended to have more surgical intervention, or alternatively have adjusted their 'normal' pain level through experiencing long periods of pain. Notably, when excluding participant smartwatch cycles after surgery for deep endometriosis, age was also strongly correlated with the mean of lower quartile 'worst' and 'average' daily fatigue values. Age was also strongly correlated with some sleep-related measures, such

as the median number of awakenings on ($R = -0.33$) as well as some physical activity measures, particularly those indicating more intense activity levels (the mean of upper quartile vigorous activity duration was correlated with age with $R = -0.43$). Examples of statistically strong correlations are displayed in Supplementary Figure 4.

There was also a notable difference in physical activity (comparing mean M10) when comparing smartwatch cycles where the smartwatch was worn on the dominant wrist compared to non-dominant. Comparisons of mean activity summary measures across participant smartwatch cycles are presented in Supplementary Figure 4; the largest difference when comparing dominant vs. non-dominant wrist was for MVPA lasting more than 10 minutes. However, these differences would primarily influence comparisons between participants, as only two participants changed wrist partway through the study and thus the wrist location would introduce minimal bias when examining changes within each individual trajectory (such as in Figure 5 of the main text). To minimize this bias, partial correlations controlling for the smartwatch wrist location were used.

(a)

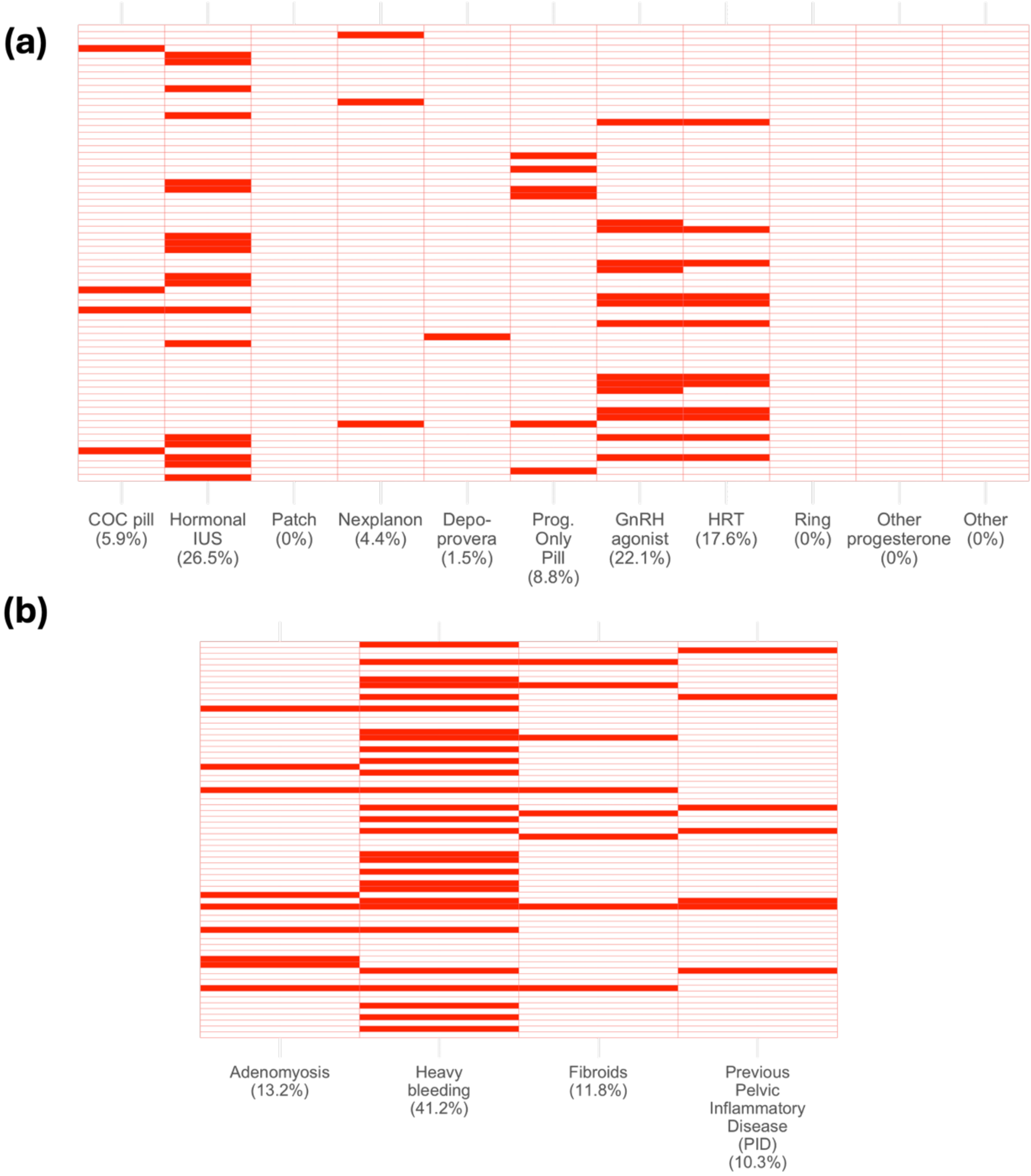

Supplementary Figure 1. Heatmap of hormonal medication and comorbidities.
A heatmap to illustrate (a) hormonal medication and (b) gynecological comorbidities by each participant (in random order), where red boxes indicate that the participant takes the medication or is diagnosed with the comorbidity.

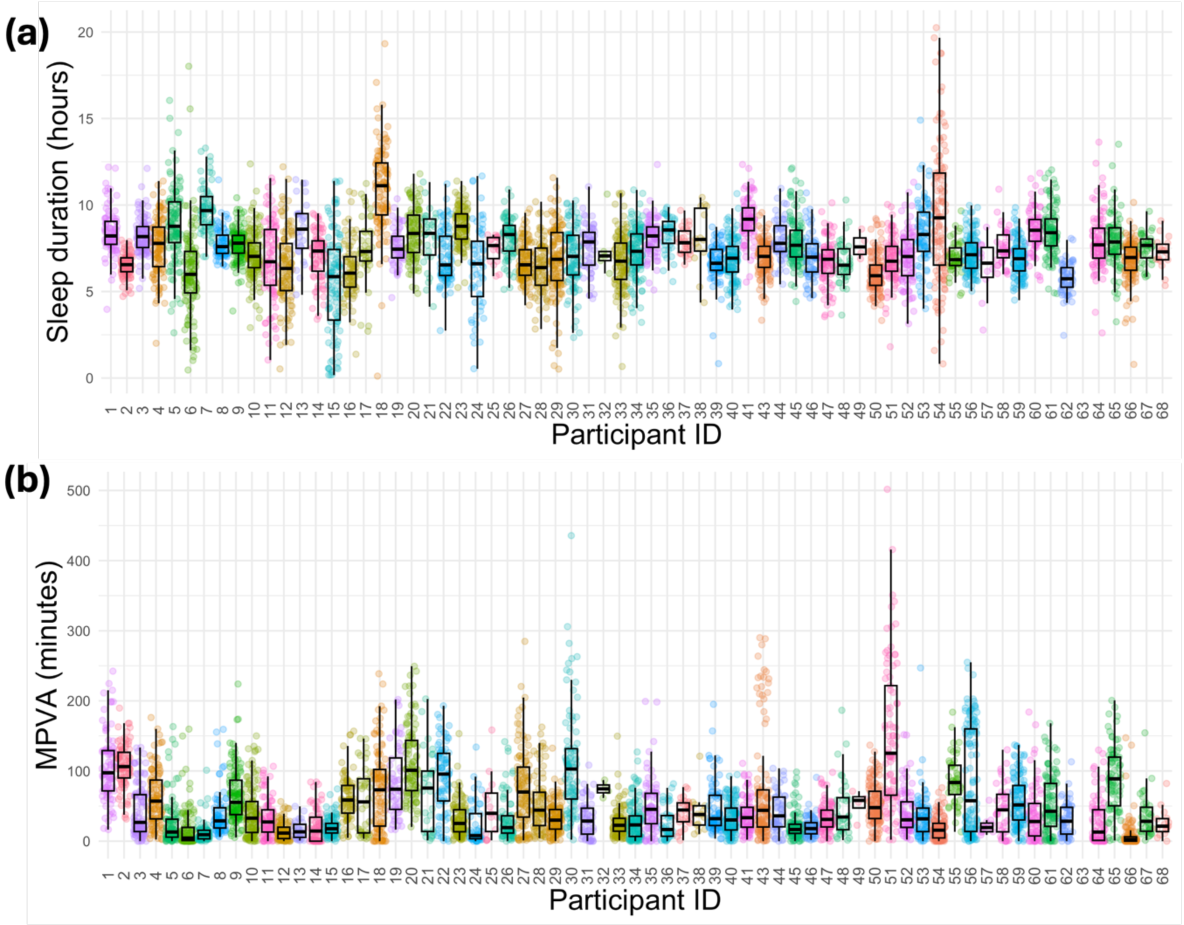

Supplementary Figure 2. Sleep duration and MVPA duration daily values by participant.

Boxplots with overlaid daily values of (a) sleep duration and (b) MVPA by participant, ordered by participant ID. Sleep duration (also referred to as sustained inactivity bouts during the night) was computed using GGIR, and excludes detected wake during the sleep period window. MVPA was also detected using the GGIR thresholds as described in the main manuscript.

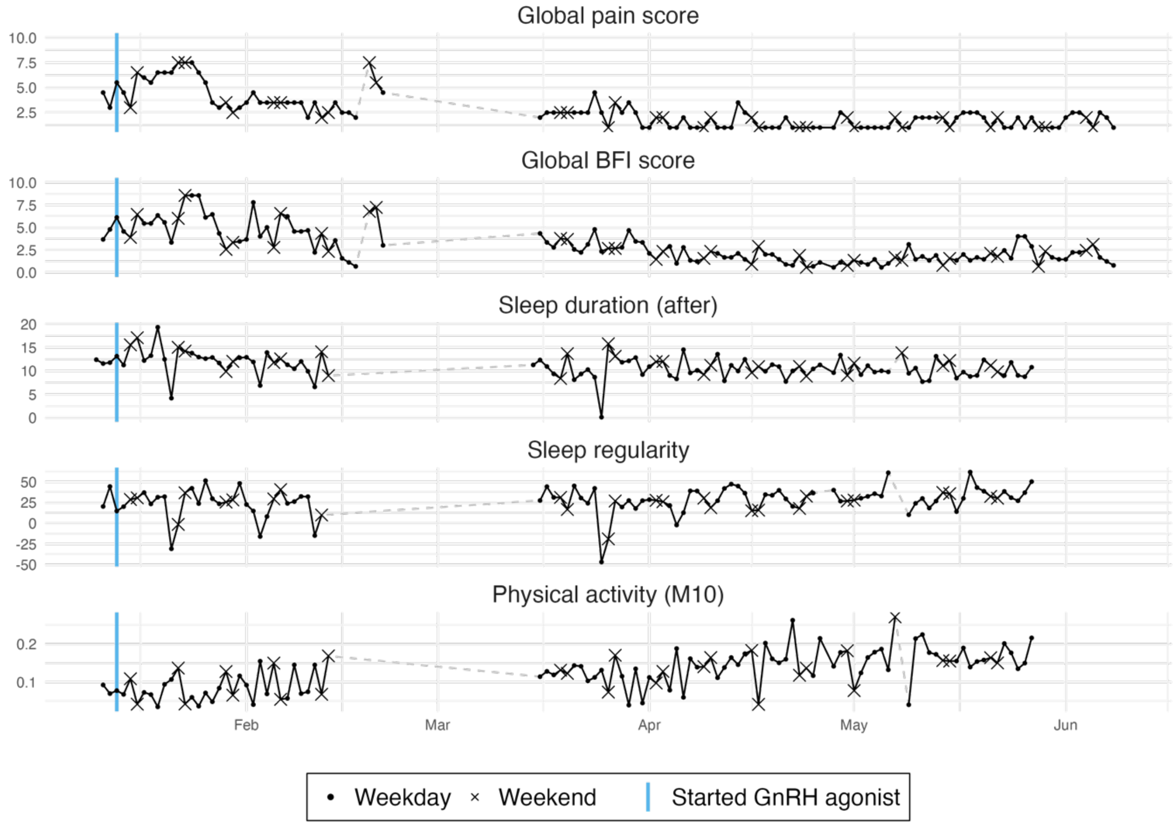

Supplementary Figure 3. Symptom trajectory for indicative participant after beginning GnRH agonist.

Here we provide an example of a participant's trajectory (participant P18) using self-reported pain and fatigue along with actigraphy-assessed measures after starting to take GnRH agonist (as indicated by the blue line) without add-back HRT. The participant's pain and fatigue decreased substantially with low variation after a few months, as the physical activity level also increased and sleep regularity increased slightly. Two other cases of participants receiving GnRH agonist before the third smartwatch cycle did not see the same decline in symptom severity, potentially due to the limited follow-up duration (4 weeks), and similarly did not see consistent improvements in physical activity or sleep in that follow-up period.

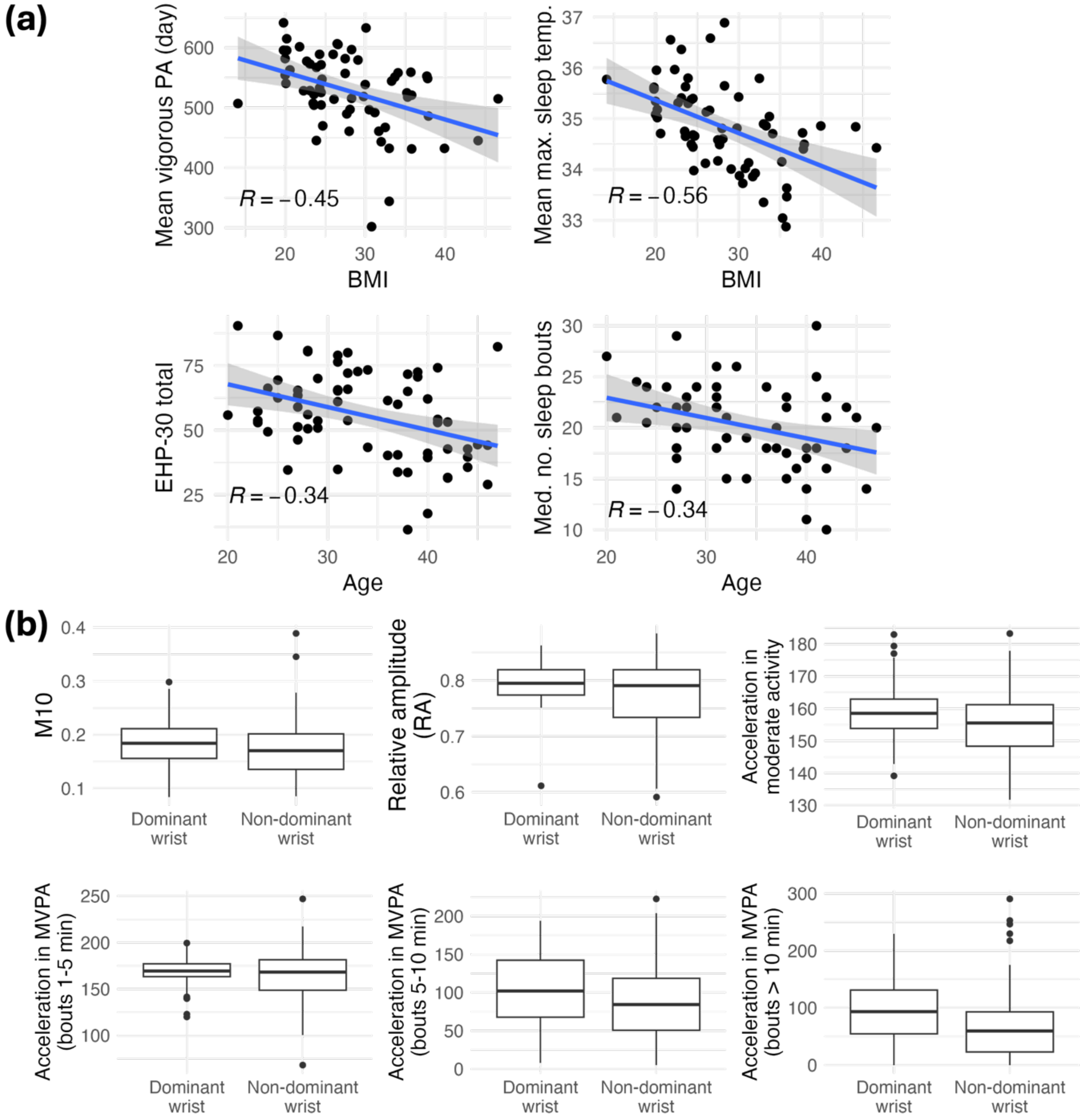

Supplementary Figure 4. Investigation of potential confounders and associations with demographic variables.

(a) Scatterplots depicting notable strong Spearman correlations between demographics (age and BMI) and measures summarized over each participant. (b) Boxplots comparing mean PA measures over each participant's smartwatch cycle by smartwatch wrist location (*N=65* smartwatch cycles in the non-dominant wrist group, and *N=112* smartwatch cycles in the non-dominant wrist group).

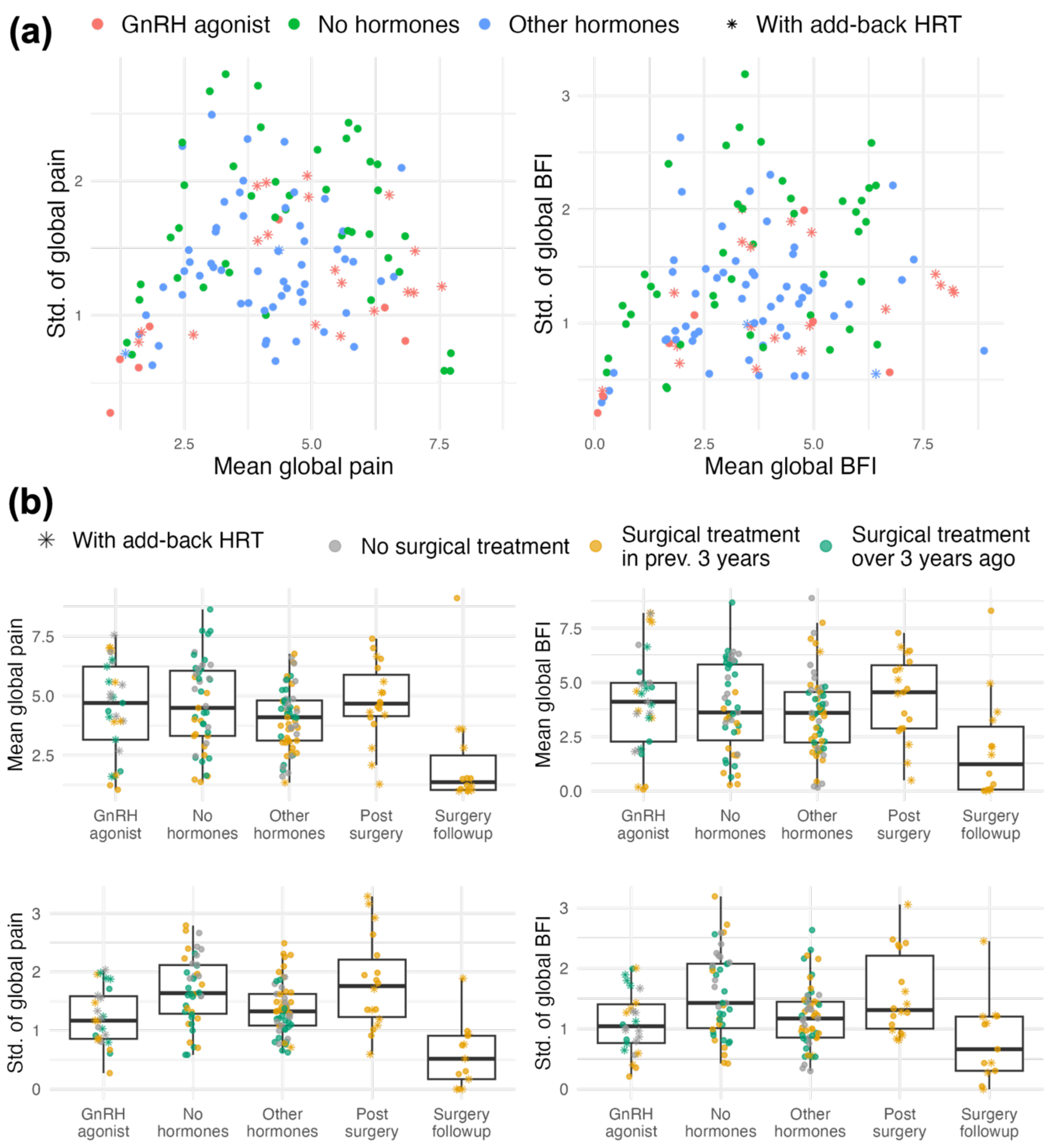

Supplementary Figure 5. Scatterplots and boxplots of cycle-level mean and standard deviation in global pain and fatigue by type of hormones taken and surgical treatment. Scatterplots comparing the mean and standard deviation in global pain and BFI scores per smartwatch cycle are shown in (a), with the color indicating whether hormones were taken, excluding smartwatch cycles where the participant had undergone surgery immediately or 4-6-months prior. The same comparison is also displayed in (b), but including smartwatch cycles completed immediately after surgery and at final follow-up (participants could also be taking hormones during these smartwatch cycles), and the color of each smartwatch cycle indicates whether the participant had received recent surgical treatment for any subtype of endometriosis (the threshold of three years is a conservative estimate based on the year of surgery as exact dates were not provided). In both figures, smartwatch cycles where add-back HRT was taken are represented by an asterisk (*). Each point represents a smartwatch cycle) over which the mean and standard deviation of global pain and global BFI scores were computed. Smartwatch cycles where a participant is taking GnRH tended to have slightly lower variability of symptoms (std.), while those taking no hormones tended to have the higher variability, particularly those that had received no surgical treatment within the previous 3 years.

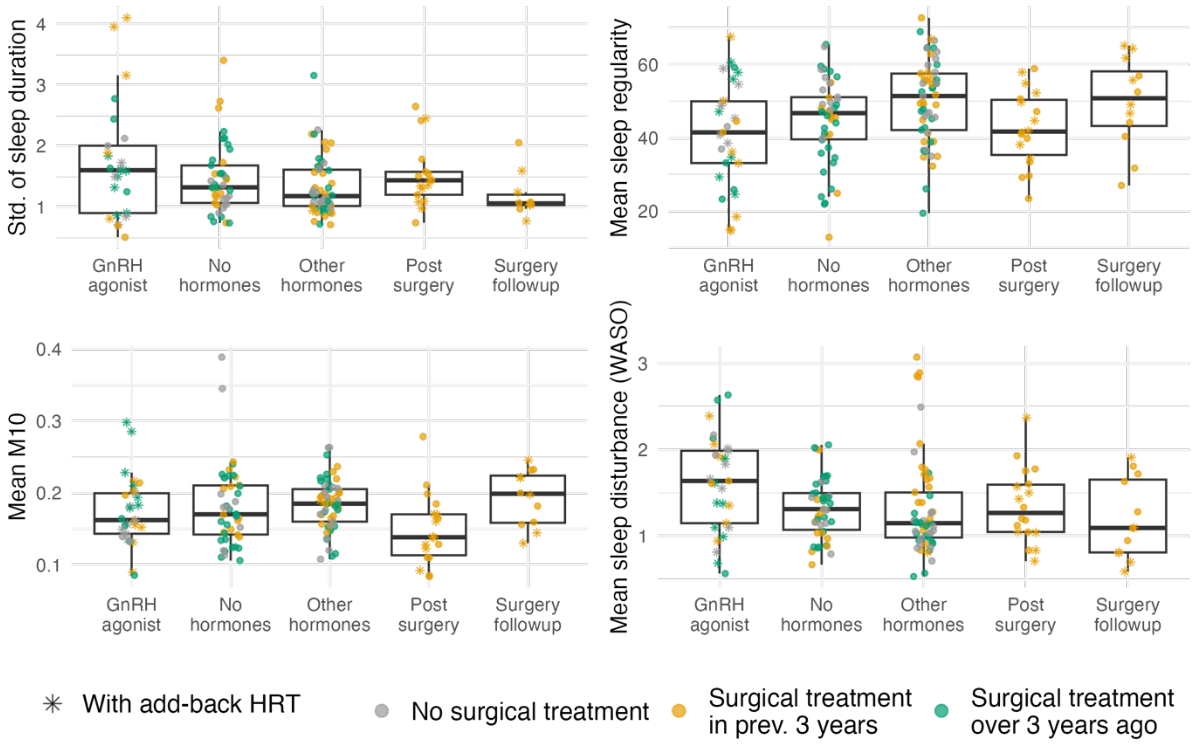

Supplementary Figure 6. Boxplots of indicative actigraphy summary measures across smartwatch cycles by hormones taken or surgical status.

Each point represents a smartwatch cycle where the actigraphy measures were summarized using mean or standard deviation. The smartwatch cycles where a participant was taking GnRH agonist tended to have more variation in sleep duration, lower sleep regularity, lower physical activity, and greater sleep disturbance. Smartwatch cycles that were completed 4-6 months following surgery or from participants that had surgical treatment within the previous three years (based on conservative estimates using the reported year of treatment) saw higher levels of physical activity and the most consistent sleep patterns.

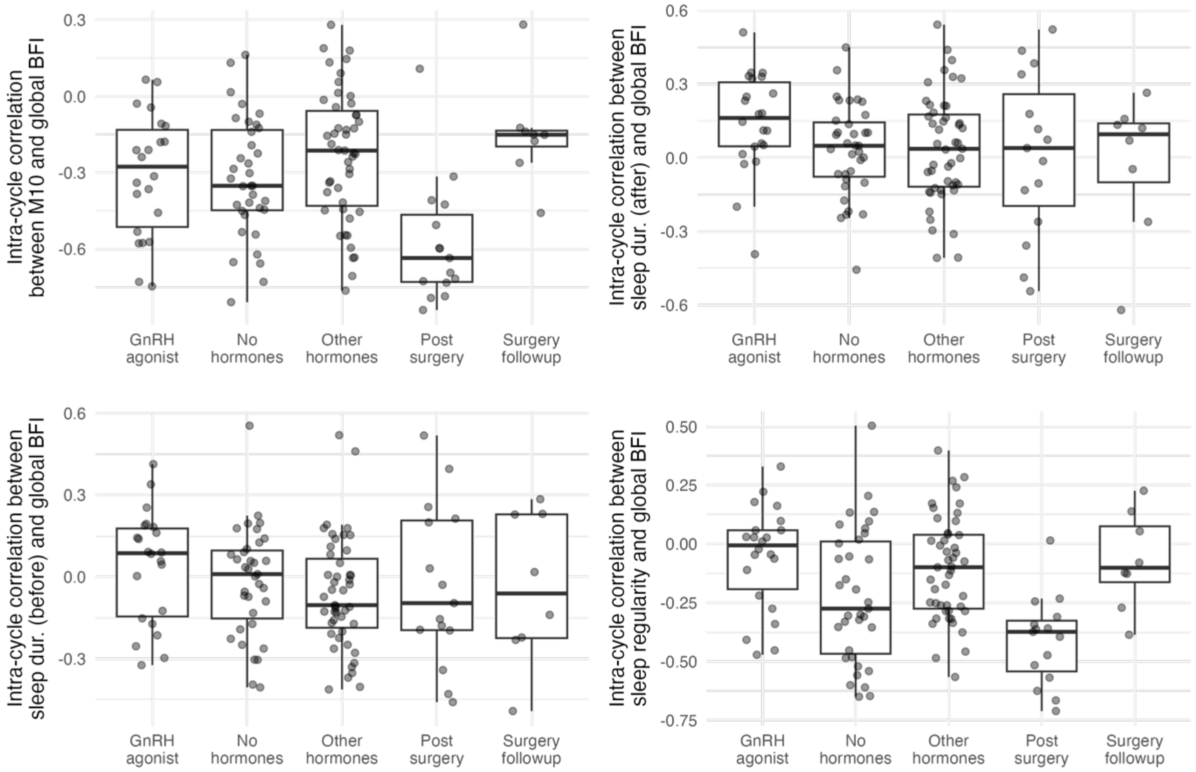

Supplementary Figure 7. Intra-cycle correlations by hormonal medication or surgical status.

A comparison of the intra-cycle (as opposed to intra-person) correlations by medication during the smartwatch cycle – where a participant is either taking GnRH agonist, no hormones, other hormonal medication, otherwise occurring immediately after surgery or 4-6 months after surgery (the latter two could also include participants also on hormones). We can see that the relationship between physical activity and fatigue is most strongly negative for smartwatch cycles immediately following surgery, whereas at follow-up there is little relationship between physical activity and fatigue, likely due to the low and less variable levels of fatigue. A similar trend is observed with sleep regularity. However, for the intra-person correlation between sleep duration (after the self-report) and fatigue, the most positive correlations were seen among the smartwatch cycles where the participant was taking GnRH agonist.

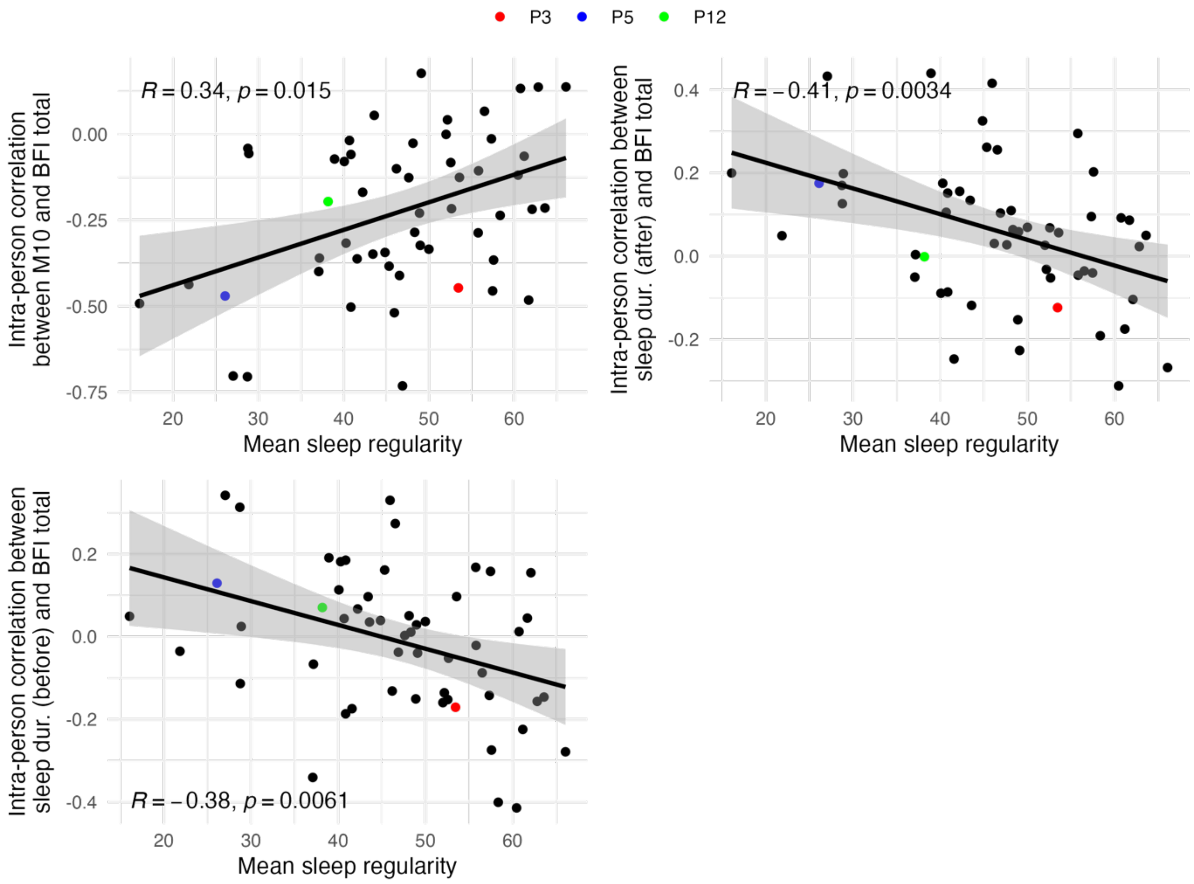

Supplementary Figure 8. Intra-person correlations compared to mean level of sleep regularity.

Here the intra-person correlations (with each data point representing a participant) between actigraphy measures and fatigue the following day are compared to mean sleep regularity for each participant. Each point represents a participant's within-person Pearson correlation coefficient. The correlations suggest that participants with highly regular sleep patterns tended to see days with lower sleep duration associated with greater fatigue, whereas participants with low sleep regularity tended to see greater sleep duration associated with fatigue. We did not find strong associations between intra-person correlations and average symptom severity, activity or sleep levels, or variation in those measures.

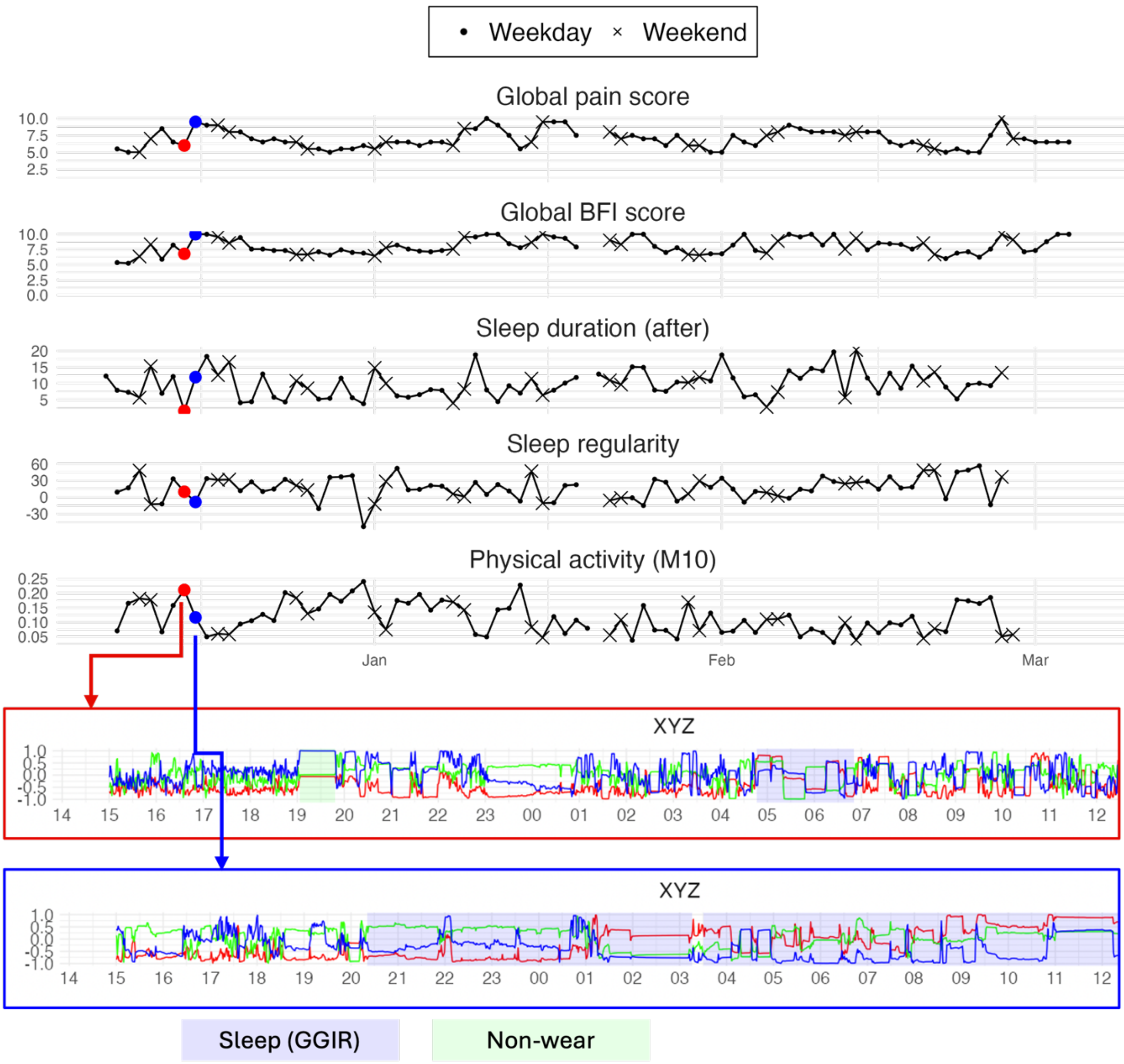

Supplementary Figure 9. Indicative example from participant P54 of variable sleep patterns and low physical activity together with severe daily symptoms.
This figure shows all three cycles from an indicative participant with abnormally long sleep detected on certain days. The top two panels show daily PROMs reported, followed by three panels showing actigraphy-assessed daily measures. The final two highlighted panels show the axis-specific acceleration values (*x, y*, and *z*) for the days indicated with arrows with detected sleep (purple) and non-wear (green) highlighted.

Supplementary Table 1. Results of linear mixed-effects models predicting daily global BFI scores and global pain scores using set of actigraphy measures.

Outcome	Predictors	Beta estimate	t-value	95% CI	p-value
Fatigue (global BFI score)	Pain (global pain score)	0.468	35.286	[0.442, 0.494]	< 0.001
	M10	-0.128	-11.953	[-0.149, -0.107]	< 0.001
	M10 (prev. day)	-0.017	-1.615	[-0.038, 0.004]	0.106
	Sleep duration	0.024	2.445	[0.005, 0.044]	0.015
	Sleep duration (prev. day)	-0.018	-1.79	[-0.038, 0.002]	0.074
	WASO	0.008	0.747	[-0.012, 0.028]	0.455
	WASO (prev. day)	0.002	0.173	[-0.018, 0.022]	0.863
	Sleep regularity index	-0.044	-3.958	[-0.066, -0.022]	< 0.001
Pain (global pain score)	Fatigue (global BFI score)	0.574	34.838	[0.542, 0.606]	< 0.001
	M10	0.014	1.195	[-0.01, 0.038]	0.251
	M10 (prev. day)	-0.019	-1.574	[-0.042, 0.004]	0.102
	Sleep duration	-0.005	-0.366	[-0.027, 0.017]	0.646
	Sleep duration (prev. day)	-0.001	0.029	[-0.023, 0.021]	0.936
	WASO	0.002	0.232	[-0.02, 0.024]	0.888
	WASO (prev. day)	0.023	2.164	[0.001, 0.045]	0.042
	Sleep regularity index	0.003	0.21	[-0.021, 0.027]	0.808

Standardized estimates of fixed effects in the mixed-effects models described in the main manuscript. Sleep duration, WASO, and sleep regularity refer to variables extracted from GGIR. Only participants with at least 20 non-missing value pairs were included, and smartwatch cycles in which participants received surgery for endometriosis (either as part of the surgical sub-study or otherwise) were excluded, resulting in 3,327 days from n=54 participants (3,265 degrees of freedom). Days with missing values for any of the variables in the model were excluded.

Supplementary Table 2. GGIR configuration parameters.

argument	value	context
config_file_in_outputdir	Config_dir	not applicable
datadir	datadir	not applicable
do.report	c(2,3,4,5)	not applicable
f0	1	not applicable
f1	172	not applicable
LC_TIME_backup	en_US.UTF-8	not applicable
mode	c(1,2,3,4,5)	not applicable
outputdir	Output_dir	not applicable
studyname	testnonwear310	not applicable
GGIRread_version	1.0.0	not applicable
GGIRversion	3.0.6	not applicable
R_version	R version 4.4.0 (2024-04-24)	not applicable
qwindow	c(0,24)	params_247
qlevels	c()	params_247
qwindow_dateformat	%d-%m-%Y	params_247
ilevels	c()	params_247
IVIS_windowsize_minutes	60	params_247
IVIS_epochsize_seconds	c()	params_247
IVIS.activity.metric	2	params_247
IVIS_acc_threshold	20	params_247
qM5L5	c()	params_247
MX.ig.min.dur	10	params_247
M5L5res	10	params_247
winhr	5	params_247
iglevels	c()	params_247
LUXthresholds	c(0,100,500,1000,3000,5000,10000)	params_247
LUX_cal_constant	c()	params_247
LUX_cal_exponent	c()	params_247
LUX_day_segments	c()	params_247
L5M5window	c(0,24)	params_247
cosinor	FALSE	params_247
part6CR	FALSE	params_247
part6HCA	FALSE	params_247
part6Window	c(start,end)	params_247
includedaycrit	16	params_cleaning
ndayswindow	7	params_cleaning

strategy		1	params_cleaning
data_masking_strategy		1	params_cleaning
maxdur		60	params_cleaning
hrs.del.start		0	params_cleaning
hrs.del.end		0	params_cleaning
includedaycrit.part5		0.66666667	params_cleaning
excludefirstlast.part5		FALSE	params_cleaning
TimeSegments2ZeroFile	c()		params_cleaning
do.imp		TRUE	params_cleaning
data_cleaning_file	c()		params_cleaning
minimum_MM_length.part5		23	params_cleaning
excludefirstlast		FALSE	params_cleaning
includenightcrit		16	params_cleaning
excludefirst.part4		FALSE	params_cleaning
excludelast.part4		FALSE	params_cleaning
max_calendar_days		0	params_cleaning
nonWearEdgeCorrection		TRUE	params_cleaning
nonwear_approach		2013	params_cleaning
segmentWEARcrit.part5		0.5	params_cleaning
segmentDAYSPTcrit.part5	c(0.9,0)		params_cleaning
study_dates_file	c()		params_cleaning
study_dates_dateformat	%d-%m-%Y		params_cleaning
overwrite		FALSE	params_general
acc.metric	ENMO		params_general
maxNcores	c()		params_general
print.filename		FALSE	params_general
do.parallel		TRUE	params_general
windowsizes	c(5,900,3600)		params_general

desiredtz	Europe/London			params_general
configtz	Europe/London			params_general
idloc			1	params_general
dayborder			0	params_general
part5_agg2_60seconds		FALSE		params_general
sensor.location	wrist			params_general
expand_tail_max_hours	c()			params_general
recordingEndSleepHour	c()			params_general
dataFormat	raw			params_general
maxRecordingInterval	c()			params_general
extEpochData_timeformat	%d-%m-%Y %H:%M:%S			params_general
do.anglex		FALSE		params_metrics
do.angley		FALSE		params_metrics
do.anglez		TRUE		params_metrics
do.zcx		FALSE		params_metrics
do.zcy		FALSE		params_metrics
do.zcz		FALSE		params_metrics
do.enmo		TRUE		params_metrics
do.lfenmo		FALSE		params_metrics
do.en		FALSE		params_metrics
do.mad		FALSE		params_metrics
do.enmoa		FALSE		params_metrics
do.roll_med_acc_x		FALSE		params_metrics
do.roll_med_acc_y		FALSE		params_metrics
do.roll_med_acc_z		FALSE		params_metrics
do.dev_roll_med_acc_x		FALSE		params_metrics
do.dev_roll_med_acc_y		FALSE		params_metrics
do.dev_roll_med_acc_z		FALSE		params_metrics
do.bfen		FALSE		params_metrics
do.hfen		FALSE		params_metrics

do.hfenplus		FALSE		params_metrics
do.lfen		FALSE		params_metrics
do.lfx		FALSE		params_metrics
do.lfy		FALSE		params_metrics
do.lfz		FALSE		params_metrics
do.hfx		FALSE		params_metrics
do.hfy		FALSE		params_metrics
do.hfz		FALSE		params_metrics
do.bfx		FALSE		params_metrics
do.bfy		FALSE		params_metrics
do.bfz		FALSE		params_metrics
do.brondcounts		FALSE		params_metrics
do.neishabouricounts		FALSE		params_metrics
hb			15	params_metrics
lb			0.2	params_metrics
n			4	params_metrics
zc.lb			0.25	params_metrics
zc.hb			3	params_metrics
zc.sb			0.01	params_metrics
zc.order			2	params_metrics
zc.scale			1	params_metrics
actilife_LFE		FALSE		params_metrics
epochvalues2csv		FALSE		params_output
save_ms5rawlevels		FALSE		params_output
save_ms5raw_format	csv			params_output
save_ms5raw_without_invalid		TRUE		params_output
storefolderstructure		FALSE		params_output
timewindow	c(MM,WW)			params_output

viewingwindow			1	params_output
dofirstpage		TRUE		params_output
visualreport		TRUE		params_output
week_weekend_aggregate.part5		FALSE		params_output
do.part3.pdf		TRUE		params_output
outliers.only		FALSE		params_output
criterror			3	params_output
do.visual		TRUE		params_output
do.sibreport		FALSE		params_output
do.part2.pdf		TRUE		params_output
sep_reports	,			params_output
sep_config	,			params_output
dec_reports	.			params_output
dec_config	.			params_output
visualreport_without_invalid		TRUE		params_output
mvpathreshold			100	params_phyact
boutcriter			0.8	params_phyact
mvpadur	c(1,5,10)			params_phyact
boutcriter.in			0.9	params_phyact
boutcriter.lig			0.8	params_phyact
boutcriter.mvpa			0.8	params_phyact
threshold.lig			40	params_phyact
threshold.mod			100	params_phyact
threshold.vig			400	params_phyact
boutdur.mvpa	c(1,5,10)			params_phyact
boutdur.in	c(10,20,30)			params_phyact
boutdur.lig	c(1,5,10)			params_phyact
frag.metrics	c()			params_phyact
part6_threshold_combi	40_100_400			params_phyact
chunksize			1	params_rawdata
spherecrit			0.3	params_rawdata
minloadcrit			72	params_rawdata
printsummary		TRUE		params_rawdata
do.cal		TRUE		params_rawdata
backup.cal.coef	retrieve			params_rawdata
dynrange	c()			params_rawdata
minimumFileSizeMB			2	params_rawdata

rmc.dec	.	params_rawdata
rmc.firstrow.acc	c()	params_rawdata
rmc.firstrow.header	c()	params_rawdata
rmc.header.length	c()	params_rawdata
rmc.col.acc	c(1,2,3)	params_rawdata
rmc.col.temp	c()	params_rawdata
rmc.col.time	c()	params_rawdata
rmc.unit.acc	g	params_rawdata
rmc.unit.temp	C	params_rawdata
rmc.unit.time	POSIX	params_rawdata
rmc.format.time	%Y-%m-%d %H:%M:%OS	params_rawdata
rmc.bitrate	c()	params_rawdata
rmc.dynamic_range	c()	params_rawdata
rmc.unsignedbit	TRUE	params_rawdata
rmc.origin	01/01/1970	params_rawdata
rmc.desiredtz	c()	params_rawdata
rmc.configtz	c()	params_rawdata
rmc.sf	c()	params_rawdata
rmc.headername.sf	c()	params_rawdata
rmc.headername.sn	c()	params_rawdata
rmc.headername.recordingid	c()	params_rawdata
rmc.header.structure	c()	params_rawdata
rmc.check4timegaps	FALSE	params_rawdata
rmc.noise	13	params_rawdata
rmc.col.wear	c()	params_rawdata

rmc.doresample		FALSE		params_rawdata
interpolationType			1	params_rawdata
imputeTimegaps		TRUE		params_rawdata
frequency_tol			0.1	params_rawdata
rmc.scalefactor.acc			1	params_rawdata
anglethreshold			5	params_sleep
timethreshold			5	params_sleep
ignorenonwear		TRUE		params_sleep
constrain2range		TRUE		params_sleep
HASPT.algo	HDCZA			params_sleep
HASIB.algo	vanHees2015			params_sleep
Sadeh_axis				params_sleep
longitudinal_axis	c()			params_sleep
HASPT.ignore.invalid		FALSE		params_sleep
loglocation	c()			params_sleep
colid			1	params_sleep
coln1			2	params_sleep
nnights	c()			params_sleep
relyonguider		FALSE		params_sleep
def.noc.sleep			1	params_sleep
sleeplogsep	c()			params_sleep
sleepwindowType	SPT			params_sleep
possible_nap_window	c(9,18)			params_sleep
possible_nap_dur	c(15,240)			params_sleep
